# A Phase Ib/II Study Evaluating the Combination of Weekly Docetaxel and Cisplatin Together with Capecitabine and Bevacizumab in Patients with Advanced Esophago-Gastric Cancer

**DOI:** 10.1371/journal.pone.0157548

**Published:** 2016-07-08

**Authors:** Baruch Brenner, Michal Sarfaty, Ofer Purim, Yulia Kundel, Limor Amit, Amir Abramovich, Udi Sadeh Gonik, Efraim Idelevich, Noa Gordon, Gal Medalia, Aaron Sulkes

**Affiliations:** 1 Institute of Oncology, Davidoff Cancer Center, Beilinson Hospital, Rabin Medical Center, Petach Tiqva, and Sackler Faculty of Medicine, Tel-Aviv University, Tel-Aviv, Israel; 2 Department of Radiology, Beilinson Hospital, Rabin Medical Center, Petach Tiqva, and Sackler Faculty of Medicine, Tel-Aviv University, Tel-Aviv, Israel; 3 Institute of Oncology, Kaplan Medical Center, Rehovot, and Hebrew Univerity, Jerusalem, Israel; ACTREC (Advanced Centre for Treatment, Research and Education in Cancer) / Tata Memorial Centre, INDIA

## Abstract

**Introduction:**

Current treatment options for advanced esophagogastric cancer (AEGC) are still unsatisfactory. The aim of this prospective phase Ib/II study was to evaluate the safety and efficacy of a novel regimen, AVDCX, consisting of weekly docetaxel and cisplatin together with capecitabine and bevacizumab, in AEGC.

**Methods:**

Patients with AEGC received treatment with different dose levels of AVDCX (cisplatin and docetaxel 25–35 mg/m^2^, days 1,8, capecitabine 1,600 mg/m^2^ days 1–14, bevacizumab 7.5 mg/kg, day 1, Q:21 days). The study's primary objectives were to establish the recommended phase II doses of docetaxel and cisplatin in AVDCX (phase Ib part) and to determine the tumor response rate (phase II part).

**Results:**

The study was closed early, after the accrual of 22 patients, due to accumulating toxicity-related deaths. The median age was 59 years and 77% of patients had gastric or gastroesophageal adenocarcinomas. Grade ≥3 adverse events were documented in 18 patients (82%), usually neutropenia (36%), fatigue (54%) or diarrhea (23%). There were three fatal toxicities (14%): mesenteric thromboembolism, gastric perforation and pancytopenic sepsis. The recommended phase II doses of cisplatin and docetaxel were determined to be 25 mg/m^2^ and 30 mg/m^2^, respectively. Twenty-one patients were evaluable for response: 12 (54%) had partial response (PR), 4 (18%) had stable disease (SD) and none had complete response (CR). Hence, the objective response rate (CR+PR) was 54% and the disease control rate (CR+PR+SD) was 72%. For the 17 patients treated at the MTD, the objective response rate was 41% and the disease control rate was 88%. The median overall survival (OS) for these patients was 13.9 months (range, 1.5–52.2 months) and the median progression-free survival was 7.6 months (range, 1.3–26.6 months). The 2-year OS rate reached 23.7%.

**Conclusions:**

AVDCX was associated with a high rate of regimen related fatal adverse events and is not appropriate for further development in AEGC patients.

**Trial Registration:**

ClinicalTrials.gov NCT00845884,

## Introduction

Gastric cancer constitutes a global health problem, representing 8.6% of the total cancer burden and the second leading cancer cause of death worldwide [1]. Chemotherapy is the mainstay of the treatment of advanced gastric cancer (AGC), offering palliation of symptoms and improved survival [2]. However, despite vigorous efforts to improve treatment efficacy, there is still no generally accepted standard regimen and the prognosis remains poor [3, 4]. Response rates of 20–40% are achieved with combination of cisplatin and 5-fluorouracil (5-FU), with a median survival of approximately 8–9 months [5, 6]. The addition of docetaxel to cisplatin/5-FU, known as the DCF regimen, showed improved response rate and overall survival (OS) in a phase III trial [7]. However, this regimen was associated with severe toxicity, including grade≥3 neutropenia in 82% of the patients and neutropenic fever in 29% [7]. As a result, DCF is still not widely accepted as a reference. The toxicity profile emphasizes the need for the development of improved DCF-like regimens which are not only more effective, but are also safer and better tolerated.

Weekly administration of docetaxel is significantly less toxic than 3-weekly administration of the drug and in most diseases where the two schedules were compared, similar efficacy was noted [8–10]. In advanced esophago-gastric cancer (AEGC), a randomized phase II trial showed improved toxicity profile with two weekly docetaxel-based regimens, both containing Cisplatin and either 5-FU or capecitabine [11]. Similar results of a phase II trial of weekly docetaxel, oxaliplatin and capecitabine, were reported in an abstract form [12].

Capecitabine, an oral prodrug of 5-FU, offers a convenient administration route obviating the need for an infuser required for protracted administration of 5-FU. Capecitabine has been consistently shown, in AEGC as well as in other diseases, to be better tolerated and at least as efficacious as intravenous 5-FU [13–17]. Furthermore, preclinical and clinical data suggest synergism of the combination of docetaxel and capecitabine, based on up-regulation of thymydine phosphorylase (TP) in tumor cells [12, 18–20].

Bevacizumab a humanized antibody against the vascular endothelial growth factor (VEGF), has shown to improve the effectiveness of standard chemotherapy in several common malignancies [21–24]. In light of encouraging results from a phase II study in AEGC [25], two phase III trials, incorporating bevacizumab into their investigational arm, were initiated in this disease [26, 27]. The results of the first phase III trial, AVAGAST, were published in 2011. In this trial, 774 patients with AGC were randomized to first-line treatment with cisplatin-fluoropyrimidine (CF), mostly capecitabine, with bevacizumab or placebo. While this trial’s results were formally negative, as the improvement in OS did not reach statistical significance, it did show improved response rate and progression free survival (PFS) in the investigational arm, and a potential impact of bevacizumab in this disease was not excluded. Moreover, an improved OS in in patients from Pan-America was suggested.

We hereby present the results of a phase Ib/II prospective trial that evaluated the safety and efficacy of a novel regimen, AVDCX, consisting of a modified DCF regimen together with bevacizumab. In this trial, all the modification described above, including the use of weekly smaller doses of docetaxel and cisplatin and the replacement of 5-FU by capecitabine, and the addition of bevacizumab, were tested in Caucasian patients with AEGC.

## Patients and Methods

This trial was a single center open-label, dose-escalation, repeat dose study, comprising two parts, Phase Ib and Phase II. Accordingly, it had two primary objectives. The primary objective of its phase Ib part was to determine the Maximum Tolerated Dose (MTD) and the recommended phase II dose of docetaxel and cisplatin in the investigational regimen. The primary objective of the phase II part was to determine the best tumor response rate (RR) until progression. The study had also several secondary objectives, including the safety profile of the AVDCX, PFS and OS, as well as patient and tumor related prognostic and predictive factors. The phase I part was designed as a 3+3 dose escalation schema as described below. The planned sample size of the phase II part was calculated according to Simon’s two-stage design, with a power of 80% and alpha of 0.05. A RR of 30% was defined as the combined null (rejecton) number for the total sample, the null hypothesis (poor) RR was 20% or less and the alternative (good) RR was 40% or higher. In the first stage, 17 patients were planned to be enrolled, including the patients in the last cohort of the phase I (receiving the phase II recommended dose). If more than three (3) patients responded in the first stage, 20 more patients were to be enrolled, for a total of 37 patients (S1 Text). Participants were recruited from 4/2009 to 06/2011, with a follow up until 12/2014.

### Patients

The study enrolled patients with histologically confirmed previously untreated metastatic or unresectable cancers of the stomach or the esophagus (AEGC), with measurable disease, no evidence of significant bleeding by the primary tumor, Eastern Cooperative Oncology Group (ECOG) performance status 0–1 and intact hematological, cardiovascular, renal and liver functions. The study protocol was approved by the Rabin Medical Center's Ethics Committee (#5294). The study was conducted in accordance with Good Clinical Practice guidelines and the Declaration of Helsinki. All patients provided written informed consent prior to study enrolment.

### Treatment and dose escalation schedule

The treatment regimen included: IV docetaxel 30 mg/m^2^ and cisplatin 30 mg/m^2^ on D1,8, IV bevacizumab 7.5 mg/kg on D1 and capecitabine 1,600 mg/m^2^ PO, divided into two daily doses, on D1-14, in 3-week cycles, on an outpatient basis.

The doses of docetaxel and cisplatin were planned to gradually increase, by 5 mg/m^2^ for each drug, to a maximum dose of 35 mg/m^2^ unless dose limiting toxicity (DLT) was observed during the first two cycles of treatment with a minimum of three subjects per cohort. If one of the first three patients in a cohort experienced DLT, three additional patients were to be treated in the same dose level. If two or more patients in the cohort experienced DLT, the next patients were to be treated with a lower dose level. In case of excessive toxicity in the starting dose level, lower dose levels were preplanned. DLT was defined as the occurrence of grade 4 or 5 hematologic toxicity, grade 3 or higher non-hematologic toxicity or any delay in treatment due to toxicity of more than one week. MTD was defined as the dose one level below the dose at which two or more patients in the cohort experienced DLT.

Patients were evaluated every two months, including history, clinical examination, complete blood count and serum chemistry and tumor assessment with a chest, abdominal, and pelvic computerized tomography (CT) scan. Response was evaluated using RECIST criteria version 1.0. Adverse events (AEs) were graded using the National Cancer Institute common toxicity criteria version 3.0 (NCI-CTCAEv3.0).

A predetermined interim safety analysis was planned when ten patients in the phase II study have completed at least two cycles of treatment. If excessive toxicity was observed the study could be modified or closed early at the discretion of the Principal Investigator.

## Results

### Patient characteristics

**Fig 1:**
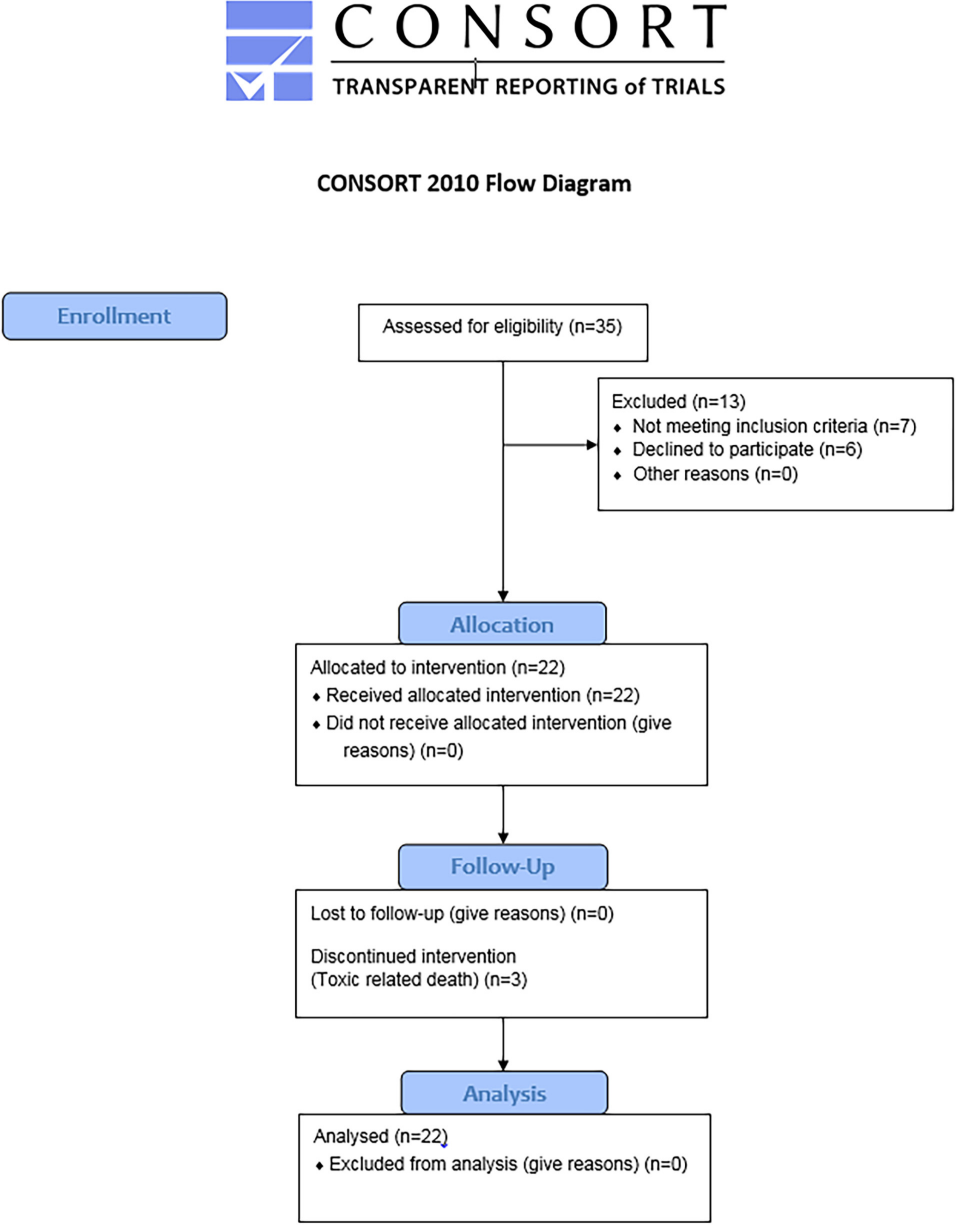
CONSORT Flow Diagram.

From 4/2009 to 06/2011, 22 patients entered this study (Fig 1). Baseline patient and tumor characteristics are shown in Table 1. The median age was 59 years (range 30–77) and 73% were males. The study population consisted predominantly (77%) of patients with gastric or gastroesophageal (GEJ) cancers. Accordingly, 91% of the tumors were adenocarcinomas or carcinomas and only 9% were squamous cell carcinomas. As the data regarding the role of trastuzumab in AGC emerged while the study was ongoing, Her2 status was not routinely tested at study entry; still, 5 patients of the 16 tested (31%) were found to have Her2 over-expressing tumors. Seventy-seven percent had more than one metastatic site and 54% had visceral involvement. Four patients (18%) had previously undergone surgery for localized disease; three of them received also adjuvant chemoradiation.

**Table 1:** Patient characteristics (n = 22).

		Number	%
Age (yr)			
	Median (range)	59 (30–77)	
Gender			
	Male	16	72.7
	Female	6	27.3
Performance status			
	0	6	27.3
	1	16	72.7
Weight loss^1^			
	Yes	17	77.3
	No	5	22.7
Disease status			
	Metastatic at presentation	18	81.8
	Metastatic at recurrence	4	18.2
Grade of differentiation			
	1–2	5	22.7
	3–4	13	59.1
	Unknown	4	18.2
Anatomical location			
	Esophagus	5	22.7
	GEJ	7	31.8
	Cardia	5	22.7
	Fundus	2	9
	Antrum	3	13.6
Histological subtype			
	Adenocarcinoma	13	59
	Mucinous/Signt Ring	5	23
	Carcinoma NOS	2	9
	Squamous cell Carcinoma	2	9
Her2 Status			
	Over-expressing	5	22.7
	Normal	11	50.0
	Unknown	6	27.3
Number of metastatic sites			
	1	5	22.7
	2	11	50.0
	3	6	27.3
Visceral metastases^2^			
	Yes	12	54.5
	No	10	45.5
Prior treatment			
	Surgery	4	18.2
	Chemotherapy	3	13.6
	Radiotherapy	3	13.6

Abbreviation: NOS = not otherwise specified.

^1^Weight loss of more than 3 kilograms.

^2^Involvment of liver and/or lungs.

### Treatment delivery

The first cohort included 3 patients who started their treatment with the initial dose (dose level 0) of cisplatin, 30 mg/m^2^. Following a DLT in that cohort, inclusion of 3 additional patients, according to the dose escalation schema, was initiated. Following a second DLT, occurring after the inclusion of two additional patients, to a total of 5 patients in the first cohort all other patients in the study started their treatment at the lower dose level (-1) of cisplatin, 25 mg/m^2^. As no other DLT's were seen, this was set as the MTD for the phase II part.

Treatment delivery is summarized in Table 2. Patients received an average of 9.6 cycles (range, 2–23), with a total of 212 cycles delivered to the entire group. The median duration of treatment was 6 months (range, 1–21), including one patient with a 4-month "drug holiday" who resumed treatment for 3 additional months after disease progression. As expected, the number of cycles in which all four drugs of the investigational regimen were delivered was smaller, with an average of 7.5 cycles per patient (range, 2–23) and a total of 166 cycles for the entire group. Dose reductions were required in 17 patients (77%); on average, the first dose reduction occurred in the 6^th^ cycle and the maximal dose reduction, i.e. the point where no further dose reductions were required, occurred in the 9^th^. While docetaxel was usually the first drug to be dose reduced, after an average of 4 months, cisplatin was usually the first drug to reach its maximal dose reduction, after an average of 4.6 months. Cisplatin was discontinued in 6 patients, mostly due to peripheral neuropathy, and Bevacizumab was discontinued in 3 patients (14%), due to uncontrolled hypertension and venous thromboembolism (VTE). Treatment delays occurred in 16 patients (73%).

**Table 2:** Treatment delivery (n = 22).

		Number	%
Cycles administered			
	Mean	9.6	
	Median	9	
	Range	2–23	
Duration of treatment (months)	Median (range)	6 (1–21)	
Cycles including all three chemotherapeutic drugs			
	Mean	7.54	
	Median	6.5	
	Range	2–15	
Dose intensity (mg/m^2^/week), mean (planned)			
	Docetaxel	28.5 (30)	95.2^1^
	Cisplatin	22.1 (30 or 25)	83.2^1^
	Capecitabine	1507.5 (1600)	94.2^1^
Dose reduction (at least once), No. of pts			
	Any drug	17	77.3
	Docetaxel	12	54.5
	Cisplatin	15	68.2
	Capecitabine	11	50.0
	Bevacizumab	0	0
Mean cycle of dose reduction			
	1^st^ dose reduction of Docetaxel	4.4	
	1^st^ dose reduction of Cisplatin	6.1	
	1^st^ dose reduction of Capecitabine	5.8	
	Maximal dose reduction of Docetaxel	8.0	
	Maximal dose reduction of Cisplatin	6.1	
	Maximal dose reduction of Capecitabine	6.7	
Treatment delay (at least once), No. of pts		16	72.7
Mean cycle of treatment discontinuation			
	Docetaxel	NR	
	Cisplatin	11.5	
	Capecitabine	NR	
	Bevacizumab	8.3	
Reason for treatment discontinuation			
	Progressive disease	19	86.3
	Toxicity	3	13.6

Abbreviations: pts = patients; NR = not relevant.

^1^Mean percentage of the individual actual vs. planned dose intensities per drug for all patients.

Despite frequent dose delays and reductions, the mean actual dose intensities of the four drugs were high, ranging between 86%-100% of the planned dose intensities. The main reason for treatment discontinuation was progressive disease (86%), followed by toxicity (14%).

### Safety

All patients were evaluable for toxicity using CTCAEv3.0 criteria. Tables 3 and 4 summarize the overall hematological and non-hematological toxicities, respectively, for the entire group and for each of the two cisplatin dose levels. Grade ≥3 AEs were documented in 18 patients (82%), and these were usually uncomplicated neutropenia, fatigue or diarrhea. The most common severe (grade ≥3) hematological toxicity was neutropenia (36%), with neutropenic fever in 9%. Growth factors were occasionally used therapeutically but not prophylactically. The most frequent severe non-hematological toxicities were fatigue (54%), diarrhea (23%), stomatitis (23%) and hand and foot syndrome (18%). Bevacizumab-related all grade toxicities were common, mainly epistaxis (54%), proteinuria (68%) and hypertension (18%), with VTE as the most common severe toxicity (14%). Toxicities were similar in the two cisplatin dose levels.

**Table 3:** Hematological toxicities^1^.

		Dose level 0 Cisplatin = 30mg/m^2^ (N = 5)		Dose level -1 Cisplatin = 25mg/m^2^ (N = 17)		All (N = 22)	
Toxicity		Grade		Grade		Grade	
		Any	≥3	Any	≥3	Any	≥3
ANC		4 (80)	2 (40)	12 (70.6)	6^#^ (35.3)	16 (72.7)	8^#^ (36.3)
	Median	1.3		1.3		1.3	
	Range	0.2–3.6		0.2–5.4		0.2–5.4	
	Fever	0 (0)		2 (11.8)		2 (9.1)	2 (9.1)
	GCSF	1 (20)		1 (5.9)		2 (9.1)	
WBC		4 (80)	2 (40)	13 (76.5)	5 (29.4)	17 (77.3)	7 (31.8)
	Median	2.68		2.89		2.78	
	Range	0.3–6.3		0.99–7.31		0.3–7.31	
Hgb		5 (100)	1 (20)	13 (76.5)	2 (11.8)	18 (81.8)	3 (13.6)
	Median	9.6		10.1		9.9	
	Range	4.7–10.6		7–13.7		4.7–13.7	
	Transfusions	1 (20)		3 (17.6)		3 (13.6)	
PLT		4 (80)	1 (20)	11 (64.7)	3 (17.6)	15 (68.2)	4 (18)
	Median	128		133		130.5	
	Range	27–201		7–278		7–278	
	Transfusions	0 (0)		3 (17.6)		3 (13.6)	

Abbreviations: ANC = absolute neutrophil count; GCSF = Granulocyte colony-stimulating factor; WBC = white blood cells; Hgb = hemoglobin.

^1^Number of patients experiencing the toxicity. Percent in parenthesis.

^#^: Grade 5 events

**Table 4:** Non-hematological toxicities (n = 22)^1^.

	Dose level 0 Cisplatin = 30mg/m^2^ (N = 5)		Dose level -1 Cisplatin = 25mg/m^2^ (N = 17)		All (N = 22)	
Toxicity	Grade		Grade		Grade	
	Any	≥3	Any	≥3	Any	≥3
Fatigue	5 (100)	3* (60)	17 (100)	9 (52.9)	22 (100)	12 (54.5)
Anorexia	3 (60)	0 (0)	4 (23.5)	1 (5)	7 (31.8)	1 (4.5)
Nausea	5 (100)	1 (20)	11 (64.7)	0 (0)	16 (72.7)	1 (4.5)
Vomiting	2 (40)	1 (20)	4 (23.5)	0 (0)	6 (27.3)	1 (4.5)
Diarrhea	4 (80)	2* (40)	12 (70.5)	3 (17.6)	16 (72.7)	5 (22.7)
Stomatitis	3 (60)	1* (20)	11 (64.7)	4 (23.5)	14 (63.6)	5 (22.7)
Neurotoxicity	2 (40)	0 (0)	9 (52.9)	0 (0)	11 (50)	0 (0)
Allergic reaction	1 (20)	1* (20)	2 (11.7)	0 (0)	3 (13.6)	1 (4.5)
Creatinine	2 (40)	0 (0)	5 (29.4)	1 (5)	7 (31.8)	1 (4.5)
Alopecia	3 (60)	0 (0)	5 (29.4)	0 (0)	8 (36.4)	0 (0)
Proteinuria	4 (80)	1 (20)	11 (64.7)	0 (0)	15 (68.2)	1 (4.5)
HFS	2 (40)	0 (0)	12 (70.5)	4 (23.5)	14 (63.6)	4 (18.2)
Hypertension	1 (20)	0 (0)	3 (17.6)	0 (0)	4 (18.2)	0 (0)
Arterial TE event	1 (20)	1*^#^	0 (0)	0 (0)	1 (4.5)	1^#^ (4.5)
Venous TE event	2 (40)	(20)	2 (11.7)	1 (5)	4 (18.2)	3 (13.6)
Perforation	0 (0)	1^#^(20)	1 (5)	1 (5)	1 (4.5)	1^#^ (4.5)
Epistaxis	3 (60)	0 (0)	9 (52.9)	0 (0)	12 (54.5)	0 (0)

Abbreviations: HFS = Hand and foot syndrome, TE = thromboembolic.

^1^Number of patients experiencing the toxicity. Percent in parenthesis.

*: DLT = Dose Limiting Toxicity event

^#^: Grade 5 events

The study was stopped early due to the occurrence of 3 fatal toxicities (14%): mesenteric event (during the phase I part), pancytopenia and sepsis and gastric perforation (during the phase II part). Hospital admissions occurred in 13 patients (59%). There were no discontinuations of treatment due to non-fatal toxicity.

### Efficacy

All but one patient, who experienced a fatal mesenteric event during the first cycle, were evaluated for response, confirmed according to RECIST. Partial response (PR) was documented in 12 patients (54%) and no complete response (CR) was noted. Four patients (18%) had stable disease (SD) for at least 9 weeks. Therefore, the objective response rate (CR+PR) was 54% and the disease control rate (CR+PR+SD) was 72%.

For the 17 patients treated at the MTD, the objective response was 41% and the disease control rate was 88%. Clinical improvement of pain was noted in 82% of these patients, weight gain in 82% and overall improvement in PS, i.e. decrease of the initial PS score, in 24%. Altogether, 14 of the 17 symptomatic patients at the initiation of AVDCX (82%) derived a clinical benefit (CB), i.e. improvement of pain, weight or PS without a deterioration of any of these factors, from treatment.

**Fig 2:**
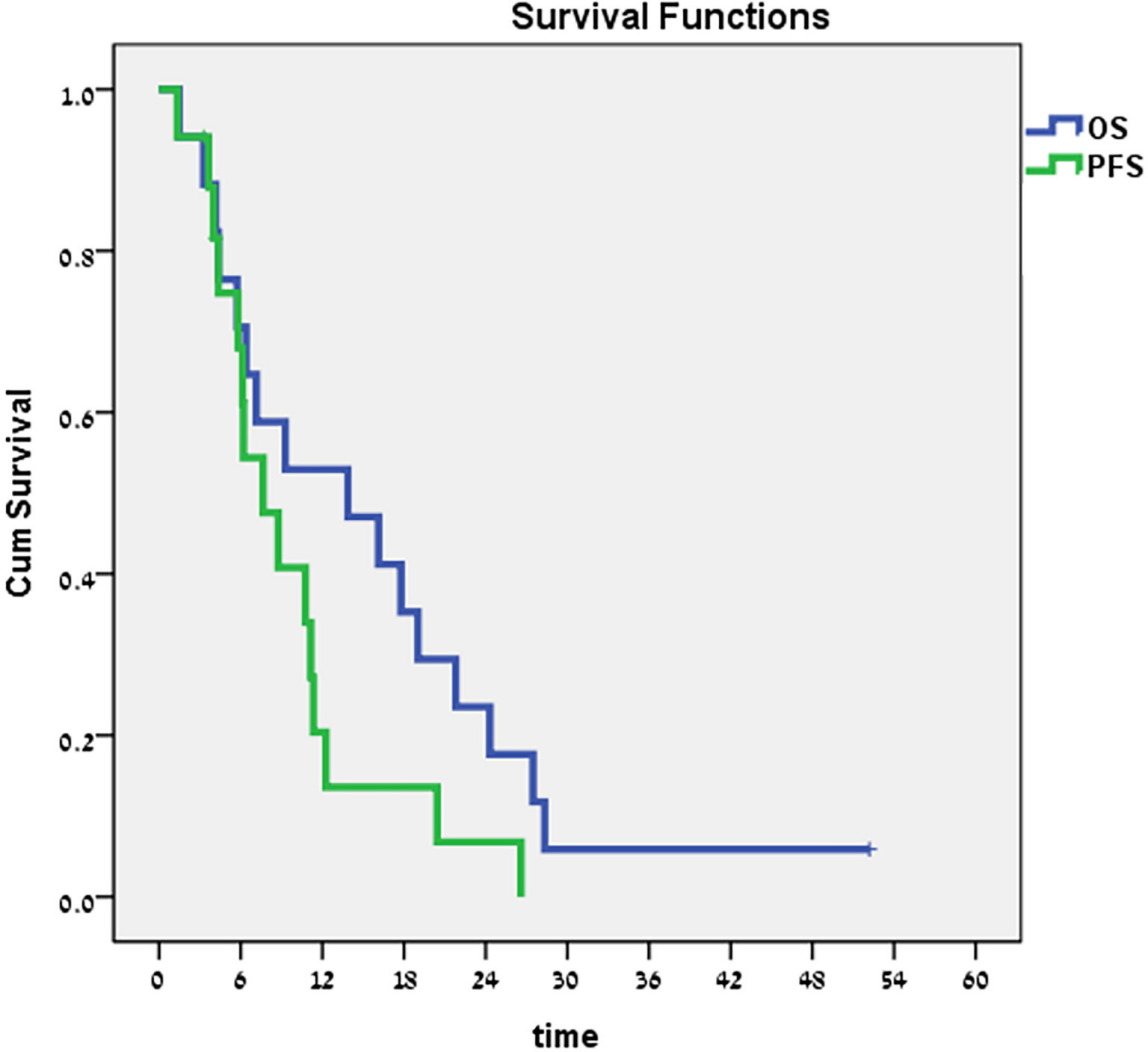
Progression-free survival (PFS) and overall survival (OS).

At the time of analysis, with a median follow-up of 22.3 months (range, 10.7–33.6), 17 patients (77%) have died of the disease, 2 (9%) are alive with disease and 3 (14%) died during treatment due to toxicity complications. For 17 patients that received the MTD, the median OS of the entire group was 13.9 months (range, 1.5–52.2 months) and the median PFS was 7.6 months (range, 1.3–26.6 months). The 2-year OS rate reached 23.7% (Fig 2).

## Discussion

This phase Ib/II prospective trial evaluated the safety and efficacy of AVDCX, a modified DCF regimen combined with bevacizumab, in Caucasian patients with AEGC. The study was closed early, after the accrual of only 22 patients, following three cases of toxicity-related deaths, two of which were related to bevacizumab therapy, resulting in a 14% mortality rate. The MTD reached was docetaxel 30 mg/m^2^ and cisplatin 25 mg/m^2^. Grade ≥3 AEs were noted in 82% of patients, the most common of which were uncomplicated neutropenia, fatigue and diarrhea. The objective response rate with the MTD was 41%, the median OS and PFS were 13.9 and 7.6 months, respectively, and the 2-year OS rate reached 23.7%.

AVDCX presented several modifications to the parent DCF regimen, aimed at improving its safety, tolerability and maybe also its anti-cancer activity. These modifications included the use of more frequent (weekly) smaller doses of docetaxel and cisplatin, replacement of IV 5-FU by oral capecitabine, and the addition of bevacizumab. While it is hard to evaluate the effect of any single modification, the overall profile of chemotherapy-related toxicities of AVDCX seems to resemble that of the original DCF [7].

Bevacizumab is known to cause hypertension, proteinuria, hemorrhage and GI perforation [28]. As opposed to previous trials, we did not observe any grade≥3 hypertension. The perforation rate in the current study (4.5%) is in accordance with the literature; unfortunately though, this single event was fatal. Thromboembolic event (TE) is a relatively common complication in cancer patients, particularly in AGC, though mostly asymptomatic [25, 29]. We experienced grade≥3 venous TEs in 13.6% and one fatal mesenteric event (4.5%). Fatal adverse events (FAEs) have been reported in 2% of cancer patients receiving bevacizumab, with increased mortality when combined with platinum or taxane [30]. Specifically in AGC, the FAEs reported in the AVAGAST trial were 2% and none in Shah's trial [25, 26]. We did not find a compelling explanation for the high 14% toxicity-related death in our study; while this may still be merely a coincidence in a small study cohort, we cannot exclude a relation to the specific combination studied.

Table 5 shows a comparison of the main patient and tumor characteristics and treatment results of our study and those of the investigational and the control (CF) arms in the DCF (V-325) and AVAGAST trials [7, 26]. In general, the patient and tumor characteristics were similar, with the exception of 29% esophageal tumors in our trial. Of all four regimens, DCF was clearly associated with the highest rates of severe toxicities, while AVDCX was associated with the highest rates of hospitalizations and treatment-related deaths. Whereas both DCF and AVDCX demonstrated the increased rate of diarrhea with the addition of docetaxel, docetaxel-associated myelosuppression was noted only with DCF. Bevacizumab-related AEs were more common in our study compared to AVAGAST, including a higher rate of FAEs. Overall, AVDCX was associated with a significantly higher rate of toxic deaths compared with the other regimens, CF, DCF and platinum/fluoropyrimidine with bevacizumab, an unexplained result of the unique combination or, more probably, the reflection of the very large impact of few events in a small sample size. Considering efficacy, DCF, AVDCX and the AVAGAST regimens resulted in similar response, PFS and OS rates, and were all clearly more efficacious than the original CF combination.

**Table 5:** Comparison between AVDCX, AVAGAST and DCF (V325) studies^1^.

		AVDCX (N = 17)	AVAGAST (N = 387)	DCF (N = 221)	CF (AVAGAST)(N = 387)	CF (V325) (N = 224)
Patient/ tumor characteristics						
	Median age (yrs)	58	58	55	59	55
	Male	76	66	72	67	71
	ECOG PS 0-1/ KPS 70–100	100	94	99	95	99
	Gastric location	71	100	100	100	100
	Metastatic disease	100	95	96	98	97
Toxicity: Grade ≥ 3						
	Any	82	76	NR	77	NR
	Neutropenia	53	35	82	37	57
	Leukopenia	29	NR	65	NR	31
	Neutropenic fever	12	5	29	4	12
	Anemia	12	10	18	14	26
	Nausea	0	7	14	10	17
	Vomiting	0	6	14	9	17
	Diarrhea	17	8	19	4	8
	Stomatitis	24	NR	21	NR	27
	Proteinuria	0	<1	NR	0	NR
	Hypertension	0	6	NR	<1	NR
	Arterial event	0	1	NR	2	NR
	Venous event	12	6	NR	0	NR
	Perforation	5	2	NR	<1	NR
	Hospitalizations	59	NR	NR	NR	NR
	Toxic deaths	6	2	2.7	3	4.5
	Discontinuation due to toxicity	0	NR	27	NR	25
Efficacy						
	Response rate	41	46	37	37.4	25
	Median PFS (months)	7.6	6.7	5.6^2^	5.3	3.7^2^
	Median OS (months)	13.9	12.1	9.2	10.1	8.6
	2-year survival	24	NR	18	NR	9

Abbreviations: AVDCX = Weekly docetaxel, cisplatin, capecitabine and bevacizumab; AVAGAST = cisplatin, fluoropyrimidine and bevacizumab DCF = docetaxel, cisplatin and 5-fluorouracil; CF = cisplatin and fluoropyrimidine; PS = Eastern Cooperative Oncology Group performance status; KPS = Karnofsky performance status; NR = not reported; PFS = progression-free survival; OS = overall survival.

^1^Percents.

^2^Time to Progression

The underlying hypothesis of our study was that the combination of several modifications of the parent DCF regimen, together with the addition of bevacizumab, will produce an improved regimen, with enhanced anti-cancer activity. Unfortunately, AVDCX seemed to be at most as active as DCF. There are several potential explanations to this result. First, most patients received a reduced dose of chemotherapy, following an early FAE, though this FAE was most probably bevacizumab and not chemotherapy related. Second, the dose intensities of both docetaxel, and especially that of cisplatin, were relatively lower than in DCF. Third, despite promising early data in AGC, weekly docetaxel was shown, in randomized trials, to be less effective than three-weekly docetaxel, at least in two other cancer types, breast [31, 32] and prostate [33] cancers. Lastly, despite the encouraging phase II data and the hints from AVAGAST in non-Asian populations, bevacizumab is probably not a very active drug in AGC, at least according to the negative primary endpoint in the phase III studies reported to date in this disease [26, 27].

In summary, this study adds to the accumulating evidence that docetaxel containing triplet regiments are clearly more effective than the CF doublet. It also validates capecitabine-based regimens as more convenient than 5-FU-based ones, with similar efficacy. In contrast with earlier phase II trials and the AVAGAST study, implying a potential benefit of bevacizumab in non-Asian patients with AEGC, we did not see any clue for such a benefit. This emphasizes the need for biomarkers or clinical characteristics which may identify subpopulations suitable for anti-angiogenic treatment strategies. We suggest close monitoring of AEGC patients receiving anti-VEGF therapy for the classic AEs, for their risk of developing life threatening complications might be higher than in other malignancies. Altogether, AVDCX was associated with unacceptable rate of fatal toxicities, with no clear suggestion for enhanced activity. Further development of this regimen is therefore unjustified.

## Supporting Information

S1 Table Patient database. (XLS)

S1 Text Study Protocol. (DOC)

S2 Text TREND statement checklist. (DOCX)
