# Supplementary material for: A Phase Ib/II Study Evaluating the Combination of Weekly Docetaxel and Cisplatin Together with Capecitabine and Bevacizumab in Patients with Advanced Esophago-Gastric Cancer
Source: PLoS One. 2016 Jul 8;11(7):e0157548. doi: 10.1371/journal.pone.0157548 (PMC4938513; doi:10.1371/journal.pone.0157548)
Supplement: S1 Text — (DOC) [file pone.0157548.s002.doc]

**CLINICAL STUDY PROTOCOL**

**A Phase I/II Study Evaluating the Combination of Weekly Docetaxel and Cisplatin Together with Capecitabine and Bevacizumab in Patients with Advanced Esophago-gastric Cancer**

| Principal Investigator & Sponsor Contact: | Dr. Baruch Brenner, M.D.[[1]](#footnote-2)  Gastrointestinal Oncology Unit Institute of Oncology, Davidiff Cancer Center Rabin Medical Center, Belinson Campus Petach Tiqva, Israel  Tel: 09-937-8005  Fax: 09-937-8045  Email: [Brennerb@clalit.org.il](mailto:Brennerb@clalit.org.il) |
| --- | --- |
| Medical Monitor: | Ms. Gal Medalia, Bsc., Institute of Oncology, Davidiff Cancer Center Rabin Medical Center, Belinson Campus |
| Clinical Laboratories: | Rabin Medical Center laboratories |
| Statistician | Dr. Jaqueline Sulkes, Rabin Medical Center, Belinson Campus |
| Date Protocol Final: | 01-OCT-2010 |
| Protocol Version | Final V3.0 |

|  |
| --- |
| **Confidentiality Statement** |
| This protocol is a confidential communication document of Dr. Baruch Brenner. The recipient of this document agrees not to disclose the information contained herein to others without prior written authorization of Dr. Baruch Brenner except that this document may be disclosed to appropriate Institutional Review Boards/Independent Ethics Committees or duly authorized representatives of Regulatory authorities such as Israeli MoH, EMEA or the US Food and Drug Administration under the condition that they maintain confidentiality. |

**Protocol Signature Page**

| Protocol Title | A phase I/II study evaluating the combination of weekly docetaxel and cisplatin together with capecitabine and bevacizumab in patients with advanced esophago-gastric cancer. |
| --- | --- |
| Protocol No. | AVDCX-2008 |
| Protocol date | 01-OCT-2010 |
| Protocol version | Final V3.0 |
| Study Phase | I/II |
| Sponsor | Dr. Baruch Brenner, investigator-initiated study. |
| Sponsor representative | Dr. Baruch Brenner Date |

By signing below, I, the investigator approve the protocol and agree to conduct the clinical trial according to all stipulations of the protocol as specified in both the clinical and administrative sections.

I agree to comply with the ICH-GCP, applicable Israeli MoH guidelines (2006) for the conduct of clinical trials, World Medical Association Declaration of Helsinki and applicable FDA regulations/guidelines set forth in 21 CFR Parts 11, 50, 54, 56, and 312.

I agree to ensure that the confidential information contained in this document will not be used for any purpose other than the evaluation or conduct of the clinical investigation without the prior written consent of Dr. Brenner.

Investigator Signature Date

Dr. Baruch Brenner Rabin Medical Center Petach Tiqva, Israel

Name Institution City, Country

###### Protocol Synopsis

| Title | A phase I/II study evaluating the combination of weekly docetaxel and cisplatin together with capecitabine and bevacizumab in patients with advanced esophago-gastric cancer. |
| --- | --- |
| Protocol No. | AVDCX-2008 |
| Clinical Site | Dr. Baruch Brenner, M.D. Gastrointestinal Oncology Unit Institute of Oncology, Davidiff Cancer Center Rabin Medical Center, Belinson Campus Petach Tiqva, Israel  Additional sites may participate in the Phase II part of the study. |
| Study Phase | I/II |
| Indication | Advanced Gastric Cancer |
| Study Objectives | The primary objective of the phase I trial is to:   - Determine the Maximum Tolerated Dose (MTD) and the recommended Phase II dose of the investigational regimen.   The secondary objective of the phase I trial is to:   - Determine the acute and subacute toxicity profile of the investigational regimen.   The primary objective of the phase II trial is to:   - Determine the tumor response rate (RR).   The secondary objectives of the phase II trial are to:   - Determine progression free survival (PFS) - Determine overall survival (OS) - Establish the safety of the recommended phase II dose. - Obtain additional data on the toxocity profile of the regimen. - Identify patient and tumor characteristics that are associated with response to the investigational regimen and to patient outcome. |
| Study Design | This will be an open-label, dose-escalation, repeat dose study comprising of two parts, Phase I and Phase II. One site will participate in the phase I study and additional sites may participate in the phase II study.  After a two-week screening phase, eligible subjects will receive combination treatment of docetaxel, cisplatin, capecitabine, and bevacizumab in 3-week cycles continuously until unacceptable toxicity, progression, or withdrawal of consent.  Each 3-week treatment cycle will consist of two infusions of docetaxel and cisplatin, one-week apart followed by one week rest, single administration of bevacizumab on Day 1, and daily administration of capecitabine from day 1 to 14, followed by one week rest. Subjects will be evaluated for safety and radiologic tumor assessment throughout the study until death.  Treatment will be given on an outpatient basis.  In the phase I component of the study, three subjects per cohort will be treated with the investigational regimen according to a dose escalation schema and will receive 14 days of treatment in each cycle unless they experience a Dose Limiting Toxicity (DLT). A DLT is defined as the occurrence of grade 4 hematologic toxicity, grade 3 or 4 non-hematologic toxicity or any delay in treatment due to toxicity of more than one week.  The dose escalation schema for the study regimen is based on the toxicities observed during the first two cycles of treatment. If in a given cohort, a DLT is observed in any of the subjects, a total of six subjects will be enrolled at the same dose level, unless a second subject experiences a DLT before all six subjects are enrolled. If less than 2 subjects in a given cohort experience a DLT, then the dose may be escalated. If a DLT is observed again, i.e. at least 2/6 subjects have a DLT, the previous dose will be considered the Maximum Tolerated Dose (MTD). The MTD is defined as the dose one level below the dose at which two (2) or more of the patients in the initial cohort experience DLT during the first two treatment courses. Upon determination of the recommended dose, the phase II part of the study will be initiated. |
| Planned Sample Size | The phase I part of the study will include up to 15 subjects. There will be 3 dose levels, with 3-6 patients per cohort.  The phase II part of the study will be conducted according to Simon’s two-stage design. In the first stage, 17 patients will be enrolled. If more than three (3) patients respond in the first stage, 20 more patients will be enrolled, for a total of 37 patients. The phase II cohort will include the patients in the last cohort of the phase I (receiving the phase II recommended dose). Therefore, the total number of patients enrolled into the two parts of the study will be 49. |
| Inclusion Criteria | 1. Patients above 18 years of age at the time of enrollment. 2. Histologically confirmed previously untreated metastatic or unresectable cancer of the esophagus or stomach. 3. Patients must have at least one measurable lesion (measuring >10mm in standard CT or >5mm in spiral CT). 4. Adequate organ function defined as:  - ANC > 1,500 cells/mm3 - Platelets > 100,000/mm3 - Hgb > 10.0 gm/dl - Serum Creatinine < 1.5 mg/dl - Creatinine Clearance Test (CCT) > 55 cc/min - Total serum bilirubin < 1.5 mg/dl  1. Life expectancy of at least three months. 2. Patients must have an ECOG Performance Status (PS) ≤ 1. 3. Signed written informed consent to participate in the study. |
| Exclusion criteria | 1. Participation in an investigational trial within 30 days of the screening visit. 2. Known allergy or any other adverse reaction to any of the study drugs or to any related compound. 3. Prior anti-angiogenic treatment, chemotherapy or radiotherapy for advanced disease. Patients will be eligible if they had received adjuvant chemotherapy or radiotherapy more than 12 months prior to enrollment. 4. Prior treatment with drugs included in the investigational regimen. Prior use of 5-fluorouracil in the adjuvant setting is allowed. 5. Significant bleeding by the primary tumor (in unoperated patients). 6. Clinically significant (i.e. active) cardiovascular disease. This includes, but is not limited to, the following examples:  - Cerebrovascular accidents (≤6 months prior to randomization) - Myocardial infarction (≤ 1 year prior to randomization). - Uncontrolled hypertension (>150/100 mmHg) while receiving chronic medication - Unstable angina - New York Heart Association (NYHA) Grade II or greater congestive heart failure. - Serious cardiac arrhythmia requiring medication. - Clinically significant ECG findings (e.g. QTc ≥ 440 msecs [male] 460 msecs [female] or ≥ 2º AV Block, etc.).   Patients who suffer from serious cardiac arrhythmia requiring medication can enter the study only if they are considered to be in a stable condition regarding both the arrhythmia and their medication. Patients with pacemakers are allowed to enter the study only if they are considered as being in a stable condition. In case of doubt, the investigator should obtain a consultation with a local cardiologist.   1. Major surgical procedure, open biopsy, or significant traumatic injury within 28 days prior to study treatment start, not fully healed wounds, or anticipation of the need for major surgical procedure during the course of the study. 2. Severe co-morbid conditions including uncontrolled diabetes or hypertension, cerebral vascular disease or uncontrolled infection. 3. Fertile subjects who are not willing to use an acceptable method of contraception during the treatment period and for 28 days following completion of treatment 4. For women of child-bearing potential: a positive pregnancy test at screening or breast-feeding. 5. History of prior malignancy (other than non-melanoma skin cancer, in-situ cervical cancer, or superficial transitional cell bladder cancer) in the last 5 years prior to enrollment. 6. Clinically significant hearing loss. 7. Patients with a history of seizure disorder who are receiving phenytoin, phenobarbital, or other antiepileptic medication. 8. Known peripheral neuropathy ≥ CTCAE v 3.0 Grade 1 9. Organ allografts requiring immunosuppressive therapy. 10. Serious, non-healing wound, ulcer, or bone fracture. 11. Evidence of bleeding diathesis or coagulopathy. 12. Current or recent (within 10 days prior to study treatment start) use of full-dose oral or parenteral anticoagulants or thrombolytic agent for therapeutic purposes. 13. Chronic, daily treatment with high-dose aspirin (> 325 mg/day), clopidogrel (> 75 mg/day). 14. Patients who cannot fully comprehend the implications of the protocol or comply with its requirements. 15. Patients with any medical or psychiatric condition or disease which, in the investigator’s judgment, would make the patient inappropriate for entry into this study 16. Presence of proteinuria at baseline. Patients discovered to have ≥2+ proteinuria on dipstick urinalysis at baseline should undergo a 24 hour urine collection and must demonstrate ≤1 g of protein in 24 hours. |
| Investigational Product Route and Dosage Form | The investigational regimen will be given in 3-week cycles. Each cycle will include a combination of:   - Docetaxel 30-35 mg/m^2^ IV, on Days 1 and 8. - Cisplatin 30-35 mg/m^2^ IV, on Days 1 and 8. - Capecitabine 1,600 mg/m^2^/d PO, divided into two daily doses, on Days 1-14. - Bevacizumab 7.5 mg/kg IV, on Day 1.   In the phase I part of the study the doses of docetaxel and cisplatin will be gradually increased, according to a predefined dose elevation schema. The initial dose level will include docetaxel 30 mg/m^2^, cisplatin 30 mg/m^2^, capecitabine 1,600 mg/m^2^ and bevacizumab 7.5 mg/kg. Once the recommended dose is determined, the phase II trial will be initiated. |
| Study Duration | The study is expected to begin on Q2/2009 and to end on Q4/ 2012 and will include approximately 3 years of recruitment period. |
| Study Procedures | **Screening Period (Day -14 to Day 0)**  The following assessments will be performed: demographic data, medical history, concurrent medications, ECOG PS, NYHA cardiac classification, physical exam, height and weight, vital signs, pregnancy test (women of childbearing potential), ECG, complete blood count (CBC), blood chemistry, CCT, tumor markers, urinalysis. Tumor assessment will include a chest, abdominal, and pelvic CT scan.  **Treatment Period (Day 1, Cycle 1, until toxicity, progression, or withdrawal of consent)**  Eligible subjects will receive IV infusion of docetaxel and cisplatin on Days 1 and 8, and bevacizumab on Day 1 of each 3-week treatment cycle. Capecitabine will be dispensed at the beginning of each cycle and will be taken per os daily for 14 days. The following assessments will be conducted at each study visit: physical examination (by a physician on Day 1 of each cycle), vital signs, ECOG PS, clinical assessment (including adverse event review and use of concomitant medications), and toxicity evaluation. Blood samples for CBC and serum electrolytes (including creatinine) will be drawn before infusion of study drugs in all treatment cycles (Days 1 and 8 of each cycle). Evaluation of blood chemistry, tumor markers and urinalysis will be done at the beginning of each cycle (Day 1 of each cycle). Tumor assessment will be done every nine weeks.  **Follow-up Period (From toxicity, progression, or withdrawal of consent to death)**  Subjects will be followed up every 2 months from the last treatment visit. Until progression, the following procedures will be done at the follow-up visit: physical exam, ECOG PS, CBC, blood chemistry, clinical assessment and tumor assessment. The tumor assessment will include a chest, abdominal, and pelvic CT scan every two months. Following progression, follow-up evaluations will focus on collecting information on further anti-tumor therapies and OS, and may be performed via phone calls.  Scheduled visits will occur at Screening, Baseline/Day 1, Day 1 of each cycle (treatment phase), and every 2 months thereafter (follow-up period). The window visit will be ±3 days for each visit during the treatment phase and ±7 days during follow up. |
| Safety Variables | - Adverse Events will be reported and graded in accordance with the National Cancer Institute Common Terminology Criteria for Adverse Events (NCI-CTCAE) version 3. - Vital signs. - Laboratory tests (CBC, chemistry, urinalysis). - Physical Examination. - Concomitant medication use. |
| Tolerability Variables | - Treatment compliance. - Vital signs. |
| Efficacy variables | - Tumor Response using RECIST criteria. - ECOG PS. |
| Statistical Methods | The safety of the study regimen will be assessed as the proportion of patients developing toxicity of any grade and those developing severe toxicities (grade >3) within 30 days following the completion of the investigational treatment. The safety analysis will include all patients who will receive at least one dose of study drugs.  PFS and OS will be calculated using the Kaplan-Meier method. OS will be calculated from registration to death or to the last date the patient was known to be alive. PFS will be calculated from registration to progression or to the last date the patient was known to be progression-free. The prognostic and predictive role of various patient and tumor characteristics will be determined as a statistically significant correlation between their presence and response to therapy (predictive factors) and patient outcome (prognostic factors). |

Protocol Synopsis [3](#__RefHeading___Toc217024860)

List of Abbreviations and Terms [13](#__RefHeading___Toc217024861)

1.0 Introduction [15](#__RefHeading___Toc217024862)

1.1 Gastric Cancer [15](#__RefHeading___Toc217024863)

1.2 Treatment of Gastric Cancer [15](#__RefHeading___Toc217024864)

1.3 Study Drugs [15](#__RefHeading___Toc217024865)

1.3.1 Cisplatin [15](#__RefHeading___Toc217024866)

1.3.2 Capecitabine [15](#__RefHeading___Toc217024867)

1.3.3 Docetaxel [16](#__RefHeading___Toc217024868)

1.3.4 Bevacizumab [16](#__RefHeading___Toc217024869)

1.4 Study Rationale [16](#__RefHeading___Toc217024870)

1.4.1 Rational for Investigational Regimen [17](#__RefHeading___Toc217024871)

2.0 Study Objectives [18](#__RefHeading___Toc217024872)

3.0 Study Design [18](#__RefHeading___Toc217024873)

4.0 Study Population [20](#__RefHeading___Toc217024874)

4.1 Inclusion Criteria [20](#__RefHeading___Toc217024875)

4.2 Exclusion Criteria [20](#__RefHeading___Toc217024876)

4.3 Subject identification [22](#__RefHeading___Toc217024877)

4.4 Removal, Replacement, or Early Withdrawal of Subjects from Therapy or Assessment [22](#__RefHeading___Toc217024878)

4.5 Handling of Withdrawals [22](#__RefHeading___Toc217024879)

4.6 Sponsor's Termination of Study [23](#__RefHeading___Toc217024880)

5.0 Study Procedures [23](#__RefHeading___Toc217024881)

5.1 Study Procedures Chart [23](#__RefHeading___Toc217024882)

5.2 Screening Visit (Day -14) [23](#__RefHeading___Toc217024883)

5.3 Baseline Visit (Day 1) [23](#__RefHeading___Toc217024884)

5.4 Treatment phase (until toxicity, disease progression or withdrawal of consent) [24](#__RefHeading___Toc217024885)

5.5 Early Discontinuation Study Visit [24](#__RefHeading___Toc217024886)

5.6 Post-Treatment Follow-Up Period (From toxicity, disease progression or withdrawal to death) [25](#__RefHeading___Toc217024887)

5.7 Treatment Compliance [25](#__RefHeading___Toc217024888)

5.8 Protocol Revisions and/or Deviations [25](#__RefHeading___Toc217024889)

6.0 Investigational Product [25](#__RefHeading___Toc217024890)

6.1 Identity of Investigational Product [25](#__RefHeading___Toc217024891)

6.2 Study Drug Administration [26](#__RefHeading___Toc217024892)

6.2.1 Parameters for treatment on Day 1 of the chemotherapy cycle [27](#__RefHeading___Toc217024893)

6.2.2 Parameters for treatment on all OTHER days (except Day 1) [27](#__RefHeading___Toc217024894)

6.3 Dose Modifications for Toxicities [27](#__RefHeading___Toc217024895)

6.4 Manufacturing [31](#__RefHeading___Toc217024896)

6.5 Packaging and Labeling [31](#__RefHeading___Toc217024897)

6.6 Distribution and Shipment [31](#__RefHeading___Toc217024898)

6.7 Storage, Dispensing and Return of the Investigational Product [31](#__RefHeading___Toc217024899)

6.8 Accountability and Compliance of Investigational Product [31](#__RefHeading___Toc217024900)

6.9 Storage of Investigational Product [32](#__RefHeading___Toc217024901)

6.10 Prior and Concomitant Therapy [32](#__RefHeading___Toc217024902)

6.10.1 General guidelines [32](#__RefHeading___Toc217024903)

6.10.2 Anticancer or experimental therapy [32](#__RefHeading___Toc217024904)

6.10.3 Anti-emetics [32](#__RefHeading___Toc217024905)

6.10.4 Hematopoetic Growth Factors [33](#__RefHeading___Toc217024906)

6.10.5 Prohibited concomitant medication [33](#__RefHeading___Toc217024907)

7.0 Assessment Procedures [33](#__RefHeading___Toc217024908)

7.1 Safety Assessments and Endpoints [33](#__RefHeading___Toc217024909)

7.1.1 Adverse events [33](#__RefHeading___Toc217024910)

7.1.2 Concomitant medication use [34](#__RefHeading___Toc217024911)

7.1.3 Treatment compliance [34](#__RefHeading___Toc217024912)

7.1.4 Vital signs [35](#__RefHeading___Toc217024913)

7.1.5 Physical Examination [35](#__RefHeading___Toc217024914)

7.1.6 Laboratory Assessments [35](#__RefHeading___Toc217024915)

7.2 Efficacy Assessment [36](#__RefHeading___Toc217024916)

7.2.1 Tumor Assessment [36](#__RefHeading___Toc217024917)

7.2.2 ECOG Performance Scale [36](#__RefHeading___Toc217024918)

8.0 Safety and Pharmacovigilance [36](#__RefHeading___Toc217024919)

8.1 Adverse Event [36](#__RefHeading___Toc217024920)

8.2 Serious Adverse Event [37](#__RefHeading___Toc217024921)

8.3 Definition of an Unexpected Adverse Event [38](#__RefHeading___Toc217024922)

8.4 Notification about Serious or Unexpected Adverse Events [38](#__RefHeading___Toc217024923)

9.0 Statistical Analysis Plan [39](#__RefHeading___Toc217024924)

9.1 Statistical Plan [39](#__RefHeading___Toc217024925)

9.2 Determination of Sample Size [39](#__RefHeading___Toc217024926)

9.3 Subject Disposition [40](#__RefHeading___Toc217024927)

9.4 Demographic and Baseline Characteristics [40](#__RefHeading___Toc217024928)

9.5 Subject Compliance [40](#__RefHeading___Toc217024929)

9.6 Concomitant Medication [40](#__RefHeading___Toc217024930)

9.7 Extent of Exposure [40](#__RefHeading___Toc217024931)

9.8 Safety Assessment [40](#__RefHeading___Toc217024932)

9.9 Efficacy Assessment [41](#__RefHeading___Toc217024933)

9.10 Interim Analysis [41](#__RefHeading___Toc217024934)

9.11 Deviation from Original Analysis Plan [41](#__RefHeading___Toc217024935)

10.0 Ethics [41](#__RefHeading___Toc217024936)

10.1 Institutional Review Board or Independent Ethics Committee [41](#__RefHeading___Toc217024937)

10.2 Ethical Conduct of the Study [41](#__RefHeading___Toc217024938)

10.3 Subject Information and Consent [42](#__RefHeading___Toc217024939)

10.4 Subject Insurance [42](#__RefHeading___Toc217024940)

10.5 Informing the General Practitioner [42](#__RefHeading___Toc217024941)

10.6 Personal Data Protection [42](#__RefHeading___Toc217024942)

10.7 Protocol Amendments [42](#__RefHeading___Toc217024943)

11.0 Quality Control and Quality Assurance [42](#__RefHeading___Toc217024944)

11.1 Study Monitoring [43](#__RefHeading___Toc217024945)

11.1.1 Source Document [43](#__RefHeading___Toc217024946)

11.1.2 Case Report Form (CRF) [43](#__RefHeading___Toc217024947)

11.2 Data Management [44](#__RefHeading___Toc217024948)

12.0 Study Administration [44](#__RefHeading___Toc217024949)

12.1 Participating Centers [44](#__RefHeading___Toc217024950)

12.2 Study Personnel [44](#__RefHeading___Toc217024951)

12.3 Required Documents Prior to Study Initiation [45](#__RefHeading___Toc217024952)

12.4 Clinical Trial Supplies [45](#__RefHeading___Toc217024953)

12.5 Investigator Site File [46](#__RefHeading___Toc217024954)

12.6 Study Completion [46](#__RefHeading___Toc217024955)

12.7 Final Report [46](#__RefHeading___Toc217024956)

12.8 Retention of Study Records [46](#__RefHeading___Toc217024957)

12.9 Confidentiality and Publication [46](#__RefHeading___Toc217024958)

13.0 References [48](#__RefHeading___Toc217024959)

APPENDICES [51](#__RefHeading___Toc217024960)

[Appendix A Study Flow Chart 52](#__RefHeading___Toc221595062)

[Appendix B Dose Escalation Scheme 53](#__RefHeading___Toc221595063)

[Appendix C Eastern Cooperative Oncology Group performance status 54](#__RefHeading___Toc221595064)

[Appendix D RECIST response criteria for solid tumors 55](#__RefHeading___Toc221595065)

[Appendix E Drug-specific treatment interruptions/discontinuations 56](#__RefHeading___Toc221595066)

[Appendix F Declaration of Helsinki 61](#__RefHeading___Toc221595067)

Tables

Table ‎3‑1 Escalation Decision Rules [3](#__RefHeading___Toc217022216)

Table ‎6‑1 Sequence of Drug Administration per Cycle [3](#__RefHeading___Toc217022217)

Table ‎6‑2 Docetaxel/CDDP/capecitabine Dose Levels [3](#__RefHeading___Toc217022218)

Table ‎6‑3 Dose Modifications according to Laboratory Results on Day 1 of the cycle [3](#__RefHeading___Toc217022219)

Table ‎6‑4 Dose Modifications according to Laboratory Results on all OTHER days [3](#__RefHeading___Toc217022220)

Table ‎6‑5 Dose Modifications for Hematological Toxicity (since the last treatment) [3](#__RefHeading___Toc217022221)

Table ‎6‑6 Dose Modifications for Non-Hematological Toxicity (since the last treatment) [3](#__RefHeading___Toc217022222)

Table ‎8‑1 Severity of Adverse Events According to CTCAE [3](#__RefHeading___Toc217022223)

Table ‎8‑2 Relationship of Adverse Event to Treatment [3](#__RefHeading___Toc217022224)

###### List of Abbreviations and Terms

| Abbreviation/Term | **Definition** |
| --- | --- |
| °C | Degrees Celsius |
| β-HCG | Beta human chorionic gonadotropin |
| 5-FU | 5-fluorouracil |
| AE | Adverse event |
| ALT | Alanine aminotransferase |
| ANC | Absolute neutrophil count |
| ASR | Age standardized rate |
| AST | Aspartate aminotransferase |
| BID | Twice a day |
| BMI | Body mass index |
| BP | Blood pressure |
| BUN | Blood urea nitrogen |
| CBC | Complete blood count |
| CCT | Creatinine clearance test |
| CDDP | *cis*-diamminedichloridoplatinum |
| CDR | Clinical data report |
| CEA | Carcino-Embryonic Antigen |
| CFR | Code of federal regulations |
| CI | Confidence interval |
| CRF | Case report form |
| CR | Complete response |
| CSF | Colony-stimulating factor |
| CT | Computed tomography |
| CTC | Common terminology criteria |
| D | Day |
| DCF | Docetaxel/CDDP/5-FU |
| dL | deciliter |
| DLT | Dose limiting toxicity |
| EC | Ethics committee |
| ECG | Electrocradiogram |
| ECOG PS | Eastern Cooperative Oncology Group Performance Status |
| EMEA | European medicines agency |
| FDA | Food & drug administration |
| GCP | Good clinical practice |
| GGT | [Gamma Glutamyl Transferase](http://encyclopedia.thefreedictionary.com/Gamma+Glutamyl+Transferase) |
| h | Hours |
| HR | Heart rate |
| ICF | Informed consent form |
| IEC | Independent ethics committee |
| IND | Investigational new drug |
| IRB | Institutional review board |
| ITT | Intent to treat |
| IV | Intravenous |
| kg | Kilogram |
| L | Liter |
| LD | Longest diameter |
| LFT | Liver function test |
| m | Meter |
| MedDRA | Medical dictionary for regulatory activities |
| mg | Milligram |
| min | Minute |
| mL | Milliliter |
| mm | Millimeter |
| MTD | Maximum tolerated dose |
| n | Number of subjects |
| NCI-CTCAE | National Cancer Institute Common Terminology Criteria for Adverse Events |
| NS | Normal saline |
| OS | Overall survival |
| PD | Progressive disease |
| PEG | Polyethylene |
| PFS | Progression free survival |
| PI | Principal investigator |
| PO | Per os |
| PR | Partial response |
| PRN | [Pro Re Nata (Latin: as needed)](http://www.thefreedictionary.com/Pro+Re+Nata) |
| PS | Performance status |
| PT | Prothrombin time (test) |
| RBC | Red blood cell |
| RECIST | Response Evaluation Criteria in Solid Tumors |
| RMC | Rabin Medical Center |
| RR | Response rate |
| SAE | Serious adverse event |
| SAP | Statistical analysis plan |
| SD | Stable disease |
| SEM | Standard error of the mean |
| TP | Thymydine phosphorylase |
| ULN | Upper limit of normal |
| USP | [United States Pharmacopeia](http://www.thefreedictionary.com/United+States+Pharmacopeia) |
| VEGF | Vascular endothelial growth factor |
| WBC | White blood cell |
| WHO | World health organization |
| μMol | Micromole |

# Introduction

## Gastric Cancer

Gastric cancer is a highly aggressive and lethal neoplasm. On a global basis, gastric cancer represents 8.6% of the entire cancer burden. In the year 2002, 933,290 new cases of gastric cancer were expected. As a reflection of its high fatality ratio, gastric cancer is the second leading cancer cause of death worldwide, with 699,802 deaths expected in 2002 [1].

Despite vigorous efforts to improve its treatment, the prognosis of gastric cancer has not changed for several decades and global five-year survival rates remain between 7-14% [2, 3]. Even after resections with curative intent, most patients will still develop locoregional and/or distant recurrence, generally within the first two years from surgery [4, 5].

## Treatment of Gastric Cancer

Chemotherapy is of crucial importance in advanced gastric cancer, in order to obtain palliation of symptoms and improve survival. The most extensively studied drugs as single agents are 5-fluorouracil (5-FU), cisplatin (CDDP), doxorubicin, epirubicin, mitomycin C and etoposide. Newer chemotherapeutic agents include the taxanes (docetaxel and paclitaxel), oral fluoropyrimidines (capecitabine and S-1), oxaliplatin and irinotecan. Randomized trials comparing monotherapy with combination regimens have consistently shown increased response rates in favor of combination regimens [6, 7].

The effectiveness of chemotherapy regimens that are currently available is limited; the most commonly used regimen, a combination of CDDP and 5-FU, is associated with a 20-40% response rate in advanced disease, with a median survival of approximately 8-9 months [8, 9]. The only clear evidence for a regimen which is more efficacious than the CDDP/5-FU combination is derived from a recent phase III trial. In this study, conducted by Van Cutsem and colleagues [10], the addition of docetaxel to CDDP/5-FU was shown to result in improved response rate and overall survival**.** However, the new regimen, known as DCF, was associated with severe toxicity: 82% of the patients had grade>3 neutropenia and 29% developed neutropenic fever [10]. As a result, DCF is still not widely accepted as a reference. This emphasizes the need for the development of newer regimens which are not only more effective than the ones that are currently used but are also safer and better tolerated.

## Study Drugs

### Cisplatin

Cisplatin (*cis*-diamminedichloridoplatinum(II), CDDP) is a [platinum](http://en.wikipedia.org/wiki/Platinum)-based [chemotherapy](http://en.wikipedia.org/wiki/Chemotherapy) [drug](http://en.wikipedia.org/wiki/Medication). Platinum complexes are formed in cells, which bind and cause cross-linking of [DNA](http://en.wikipedia.org/wiki/DNA), ultimately triggering [apoptosis](http://en.wikipedia.org/wiki/Apoptosis), or automated cell death [11]. Cisplatin is a widely used anticancer agent with a broad range of antitumor activities, including against gastric cancer [12].

### Capecitabine

Capecitabine (Xeloda^TM^) is a new fluoropyrimidine carbamate prodrug, which is designed to be sequentially converted to 5-fluorouracil (5-FU) by three enzymes located in the liver and in tumors; the final step is the conversion of 5'-deoxy-5-fluorouridine (5'-DFUR) to 5-FU by thymidine phosphorylase in tumors [13].

Capecitabine has been consistently shown, in colon and breast cancers, to be better tolerated and at least as efficacious as intravenous 5-FU [14-16]. A recently reported phase III trial in gastric cancer has demonstrated that capecitabine can safely replace 5-FU in the epirubicin/CDDP/5-FU (ECF) regimen [17].

### Docetaxel

Docetaxel (Taxotere^TM^) is an antineoplastic agent of the taxoid family that acts by disrupting the microtubular network in cells that is essential for mitotic and interphase cellular functions [18].

Docetaxel is widely used against various malignancies, especially breast and lung cancers [19-21]. Cumulative data suggest that the drug is also very active against gastric cancer. In a randomized phase II trial conducted by Roth and colleagues [22] the combination of docetaxel, CDDP and 5-FU (TCF) was shown to result in increased response rate compared with docetaxel and 5-FU alone (TC) or the standard ECF regimen [22]. A phase III clinical trial, conducted by Van Cutsem and colleagues [10], included 445 patients and has shown docetaxel to improve survival of patients with advanced gastric cancer. Patients treated with the docetaxel-based chemotherapy regimen, including docetaxel, CDDP, and 5-FU (DCF) experienced a significant 23 percent reduction in the risk of death compared to patients who received CDDP and 5-FU alone. The median overall survival and time to disease progression were significantly longer with the docetaxel-containing regimen (9.2 vs. 8.6 months, p=<0.02, and 5.6 vs. 3.7 months, p=0.0004, respectively) [10]. Based on these results, the U.S. Food and Drug Administration (FDA) has approved docetaxel for use in combination with CDDP and 5-FU for the treatment of patients with advanced gastric adenocarcinoma who have not received prior chemotherapy for advanced disease.

### Bevacizumab

Bevacizumab (Avastin^TM^) is a humanized antibody against the vascular endothelial growth factor (VEGF). VEGF is a key molecule in both tumor angiogenesis and the survival of tumor endothelial cells [23].

Bevacizumab was shown to improve the results of standard chemotherapy in several common malignancies [24-27]. The drug was approved by the FDA for the treatment of metastatic colorectal cancer, advanced non-small cell lung cancer and metastatic HER2-negative breast cancer in combination with chemotherapeutic drugs. In a recent phase II study conducted by Shah and colleagues [28], 47 patients with metastatic or unresectable gastric adenocarcinoma were treated with combination of bevacizumab, irinotecan, and cisplatin. Overall, response rate was 65%, median survival was 12.3 months (95% CI, 11.3 to 17.2 months), and time to tumor progression was improved by 75% compared with historical controls. No increase in chemotherapy related toxicity was observed with this regimen [28]. In light of these encouraging results, several ongoing phase III trials in gastric cancer are incorporating bevacizumanb into their investigational arm.

## Study Rationale

In spite of multiple attempts to improve the efficacy of first-line chemotherapy in advanced gastric cancer, the progress that has been achieved so far is rather limited; for example, in the positive pivotal phase III trial, leading to the registration of decetaxel for advanced gastric cancer, the impact on overall survival was marginal, with an absolute difference in median survival of 0.6 month (9.2 vs 8.6 months [10]. Even this modest advance was associated with significant cost of toxicity which avoided the general adaptation of the DCF regimen [10]. Clearly, there is an urgent need for novel regimens which are more efficacious and less toxic than the current ones.

### Rational for the Investigational Regimen

A modified DCF regimen, which would have at least equivalent efficacy and an improved toxicity profile could become the standard of care in advanced gastric cancer. The proposed regimen has a potential to be at least as effective as and less toxic than DCF, for the following reasons:

1. Weekly administration of docetaxel is significantly less toxic than 3-weekly administration of the drug and in most diseases where the two schedules were compared similar efficacy was noted [19, 20, 29]. In esophago-gastric cancer, a recently completed randomized phase II trial showed improved toxicity profile with two weekly docetaxel-based regimens, both containing CDDP and either 5-FU or capecitabine [30]. Similar results were noted in a phase I trial of weekly docetaxel, oxaliplatin and capecitabine [31].
2. Oral administration of capecitabine in combination with CDDP has a potential for an improved therapeutic index compared with CDDP/5-FU in gastric cancer patients [17, 32, 33]. The safety and efficacy of the capecitabine/CDDP regimen in gastric cancer patients are supported by the results of phase II and phase III trials [34, 35]. In a phase III trial, a capecitabine/CDDP regimen resulted in superior response rate compared to a CDDP/ 5-FU regimen (41% vs. 29%, p=0.003, respectively) and had a more favorable toxicity profile [35].

Furthermore, in breast cancer, the combination of docetaxel and capecitabine was shown to be synergistic and to improve patient outcome [21]. These clinical results are supported by preclinical evidence for synergism, based on up-regulation of thymydine phosphorylase (TP), the third and final activating enzyme of capecitabine (and the one responsible for the relative high intracellular levels of the drug in tumor cells), in tumor cells, following exposure to docetaxel [36]. Clues for a similar effect were noted in several phase II trials in gastric cancer too [31, 37].

1. Bevacizumab has the potential to improve the efficacy of the chemotherapy regimen since there is preclinical evidence that the VEGF pathway is vital in gastric cancer [38, 39]. Phase II studies have shown encouraging results with the addition of bevacizumab to the treatment of advanced gastric cancer [28, 40] and randomized phase III trials evaluating the drug in this setting are ongoing. Bevacizumab is generally well tolerated, with a toxicity profile which does not overlap with those of conventional chemotherapy. Therefore, while the addition of bevacizumab may enhance the efficacy of the cisplatin/capecitabine/docetaxel combination, the overall toxicity of the combined regimen is not anticipated to increase substantially.

The goal of this two-part, phase I/II, open label study is to investigate the efficacy and safety of cisplatin/capecitabine/docetaxel/bevacizumab combination treatment in up to 52 patients with advanced gastric cancer. Results will be compared to the data in the literature.

# Study Objectives

The primary objective of the phase I trial is to:

- Determine the Maximum Tolerated Dose (MTD) and the recommended Phase II dose of the investigational regimen.

The secondary objective of the phase I trial is to:

- Determine the acute and subacute toxicity profile of the investigational regimen.

The primary objective of the phase II trial is to:

- Determine the tumor response rate (RR).

The secondary objectives of the phase II trial are to:

- Determine progression free survival (PFS)
- Determine overall survival (OS)
- Establish the safety of the recommended phase II dose.
- Obtain additional data on the toxocity profile of the regimen.
- Identify patient and tumor characteristics that are associated with response to the investigational regimen and to patient outcome.

# Study Design

This will be an open-label, repeat dose study comprised of two parts, phase I and II, in subjects with advanced esophago-gastric cancer. The phase I study will be conducted in a single site, the Rabin Medical Center (RMC) but additional sites may participate in the phase II study

After a two-week screening phase, eligible subjects will receive combination chemotherapy with docetaxel, cisplatin, capecitabine, and bevacizumab in 3-week cycles continuously until unacceptable toxicity, disease progression or patient's withdrawal of consent. Temporary discontinuation of treatment ("drug holiday"), until disease progression, will be allowed after a minimum of six months of treatment.

Treatment will be given in an outpatient setting. Each 3-week treatment cycle will consist of two infusions of docetaxel and cisplatin, one-week apart (days 1 and 8) followed by one week rest, a single administration of bevacizumab on day 1, and daily administration of capecitabine from day 1 to 14, followed by one week rest. On day 1 of each cycle, the sequence of drug administration will be as follows: bevacizumab will be administered first, followed by docetaxel and then by cisplatin. On that day, the morning dose of capecitabine will be taken at the time of premedication (prior to all other agents). On day 8 of each cycle, the same sequence will take place but no bevacizumab will be given. Subjects will be evaluated for safety and radiologic tumor assessment throughout the study until death.

The study will be composed of two parts:

**Part A – Phase I Study**

This will be a phase I, single-center, open-label, repeat dose, dose escalation study. There will be three (3) dose levels, with 3-6 patients per cohort. A minimum of three subjects per cohort will be treated with the investigational regimen using escalating doses of docetaxel and cisplatin; the doses of capecitabine and bevacizumab will not be escalated. The doses of docetaxel and cisplatin will be gradually increased in each consecutive cohort, according to a predefined dose escalation schema (Appendix B). Patients will receive 14 days of treatment in each cycle unless they experience a Dose Limiting Toxicity (DLT). A DLT is defined as the occurrence of grade 4 hematologic toxicity, grade 3 or 4 non-hematologic toxicity or any delay in treatment due to toxicity of more than one week.

The Maximum Tolerated Dose (MTD) is defined as the dose one level below the dose at which two (2) or more of the patients in the initial cohort experience DLT during the first two treatment cycles. Upon determination of the recommended dose, the phase II part of the study will be initiated.

Table ‎3‑1 Escalation Decision Rules1

| **Number of Patients with DLT at a Given Dose Level** | **Escalation Decision Rule** |
| --- | --- |
| 0 out of 3 | Enter 3 patients at the next dose level. |
| >2 | Dose escalation will be stopped. This dose level will be declared the MTD. Three (3) additional patients will be entered at the next lowest dose level if only 3 patients were treated previously at that dose. |
| 1 out of 3 | Enter at least 3 more patients at this dose level.   - If 0 of these 3 patients experience DLT, proceed to the next dose level. - If 1 or more of this group suffer DLT, then dose escalation is stopped, and this dose is declared the MTD. Three (3) additional patients will be entered at the next lowest dose level if only 3 patients were treated previously at that dose. |
| <1 out of 6 at highest dose level below the maximally administered dose | This is the recommended phase 2 dose. At least 6 patients must be entered at the recommended phase 2 dose. |

^1*^From NCI CTEP Phase I protocol template ([http://ctep.cancer.gov/guidelines/templates.html](http://www.pubmedcentral.nih.gov/redirect3.cgi?&&auth=061E2jePas-xoFW3k2DEmi_nU4vMqwVMSfh1E-kzp&reftype=extlink&artid=1885070&iid=144705&jid=279&FROM=Article|Body&TO=External|Link|URI&article-id=1885070&journal-id=279&rendering-type=normal&&http://ctep.cancer.gov/guidelines/templates.html)).
 Abbreviations: MTD, Maximum tolerated dose; DLT, dose-limiting toxicity.

**Part B** **– Phase II study**

This will be a phase II, open-label, repeat dose study. The study will be conducted according to Simon’s two-stage design. In the first stage, 17 patients will be enrolled. If more than three (3) patients respond in the first stage, 20 more patients will be enrolled, for a total of 37 patients. The phase II cohort will include the patients in the last cohort of the phase I (receiving the phase II recommended dose).

**Summary of Study Procedures for Phase I and II**

In both study parts, after providing written informed consent, subjects will be screened for good general health, lack of evidence of other diseases, and pregnancy (women only) amongst other criteria for inclusion. If eligible for the study, subjects will be enrolled. Below is a brief summary of the screening, treatment and follow-up periods.

**Screening (Day -14 to Day 0)**

The following assessments will be performed: demographic data, medical history, ECOG performance status (PS), NYHA cardiac classification, physical exam, height and weight, vital signs, pregnancy test (women of childbearing potential), ECG, complete blood count (CBC), blood chemistry, creatinine clearance test (CCT), tumor markers (CEA, CA-19.9 and CA-125) and urinalysis. A clinical assessment including adverse event (AE) review and concurrent medication use will also be done. The tumor assessment will include a chest, abdominal, and pelvic CT scan for all subjects.

**Treatment Phase (Day 1, Cycle 1 until toxicity, disease progression, or withdrawal of consent)**

Eligible subjects will receive IV infusion of docetaxel and cisplatin on Days 1 and 8 and bevacizumab on Day 1 of each 3-week treatment cycle. Capecitabine will be dispensed at the beginning of each cycle and will be taken daily for 14 days. The following assessments will be conducted at each study visit: physical examination (by a physician on Day 1 of each cycle), vital signs, ECOG PS, clinical assessment (including AE review and use of concomitant medication), and toxicity evaluation. Blood samples for CBC and serum electrolytes (including creatinine) will be drawn before infusion of study drugs in all treatment cycles (Days 1 and 8 of each cycle). Evaluation of blood chemistry, tumor markers (if elevated at baseline) and urinalysis will be done at the beginning of each cycle (Day 1 of each cycle). Tumor assessment will be done every nine (9) weeks using the RESCIST criteria.

**Follow-up Period (From toxicity, disease progression, or withdrawal of consent to death)**

Subjects will be followed every two (2) months from the last treatment visit. The following procedures will be done at the follow-up visit: physical exam, ECOG PS, CBC, blood chemistry, clinical assessment and tumor assessment. The tumor assessment will include a chest, abdominal, or pelvic CT scan every three months for all subjects. This relates also to patients having a temporary break in their treatment ("drug holiday").

Scheduled visits will occur at Screening, Baseline/Day 1, Days 1 and 8 of each cycle (treatment phase), and every two (2) months thereafter (follow-up period). The window visit will be ± 3 days for each visit during the treatment phase and ± 7 days for the follow up period.

# Study Population

Subjects above 18 years of age with advanced esophago-gastric cancer will be included in this study. Up to 15 subjects will participate in the phase I component of the study and up to 37 subjects will participate in its phase II component. Patients who were treated in the last cohort of the phase I part of the study (i.e. received the phase II recommended dose) will be included in these 37 patients.

## Inclusion Criteria

Subjects must meet all inclusion criteria in order to be eligible for the study:

1. Patients above 18 years of age at the time of enrollment.
2. Histologically confirmed previously untreated metastatic or unresectable cancer of the esophagus or stomach.
3. Patients must have at least one measurable lesion (measuring >10mm in standard CT or >5mm in spiral CT).
4. Adequate organ function defined as:

ANC > 1,500 cells/mm^3^

Platelets > 100,000/mm^3^

Hgb > 10.0 gm/dl

Serum Creatinine < 1.5 mg/dl

Creatinine Clearance Test (CCT) > 55cc/min

Total serum bilirubin < 1.5 mg/dl

1. Life expectancy of at least three (3) months.
2. Patients must have an ECOG PS ≤ 1.
3. Signed written informed consent to participate in the study.

## Exclusion Criteria

Any of the following conditions will exclude the subject from entering the study:

1. Participation in an investigational trial within 30 days of the screening visit.
2. Known allergy or any other adverse reaction to any of the study drugs or to any related compound.
3. Prior anti-angiogenic treatment, chemotherapy or radiotherapy for advanced disease. Patients will be eligible if they had received adjuvant chemotherapy or radiotherapy more than 12 months prior to enrollment.
4. Prior treatment with drugs included in the investigational regimen. Prior use of 5-FU in the adjuvant setting is allowed.
5. Significant bleeding by the primary tumor (in unoperated patients).
6. Clinically significant (i.e. active) cardiovascular disease**.** This includes, but is not limited to, the following examples:
   - Cerebrovascular accidents (< 6 months prior to randomization).
   - Myocardial infarction (≤ 1 year prior to randomization).
   - Uncontrolled hypertension (>150/100 mmHg) while receiving chronic medication.
   - Unstable angina.
   - New York Heart Association (NYHA) Grade II or greater congestive heart failure.
   - Serious cardiac arrhythmia requiring medication.
   - Clinically significant ECG findings (e.g. QTc ≥ 440 msecs [male] 460 msecs [female] or ≥ 2º AV Block, etc.).

Patients who suffer from serious cardiac arrhythmia requiring medication can enter the study only if they are considered to be in a stable condition regarding both the arrhythmia and their medication. Patients with pacemakers are allowed to enter the study only if they are considered as being in a stable condition. In case of doubt, the investigator should obtain a consultation with a local cardiologist.

1. Major surgical procedure, open biopsy, or significant traumatic injury within 28 days

prior to study treatment start, not fully healed wounds, or anticipation of the need for

major surgical procedure during the course of the study. Central venous access device (CVAD) for chemotherapy administration must be inserted at least one day prior to treatment start.

1. Severe co-morbid conditions including uncontrolled diabetes or hypertension, cerebral vascular disease or uncontrolled infection.
2. Fertile subjects who are not willing to use an acceptable method of contraception during the treatment period and for 28 days following completion of treatment
3. For women of child-bearing potential: a positive pregnancy test at screening or breast-feeding.
4. History of prior malignancy (other than non-melanoma skin cancer, in-situ cervical cancer, or superficial transitional cell bladder cancer) in the last five years prior to enrollment.
5. Clinically significant hearing loss.
6. Patients with a history of seizure disorder who are receiving phenytoin, phenobarbital, or other antiepileptic medication.
7. Known peripheral neuropathy ≥ CTCAE v 3.0 Grade 1. Absence of deep tendon reflexes as the sole neurological abnormality does not render the patient ineligible.
8. Organ allografts requiring immunosuppressive therapy.
9. Serious, non-healing wound, ulcer, or bone fracture.
10. Evidence of bleeding diathesis or coagulopathy.
11. Current or recent (within 10 days prior to study treatment start) use of full-dose oral

or parenteral anticoagulants or thrombolytic agent for therapeutic purposes.

1. Chronic, daily treatment with high-dose aspirin (> 325 mg/day), clopidogrel (> 75 mg/day).
2. Patients who cannot fully comprehend the implications of the protocol or comply with its requirements.
3. Patients with any medical or psychiatric condition or disease which, in the investigator’s judgment, would make the patient inappropriate for entry into this study.
4. Presence of proteinuria at baseline. Patients discovered to have ≥2+ proteinuria on dipstick urinalysis at baseline should undergo a 24 hour urine collection and must demonstrate ≤1 g of protein in 24 hours.

## Subject identification

A unique subject number will be assigned when an individual subject is qualified for study enrollment. The subject will receive study drugs from the next available subject package. Before entry, subjects will be identified by their initials and birth date only.

## Removal, Replacement, or Early Withdrawal of Subjects from Therapy or Assessment

Subjects are free to discontinue their participation in the study at any time and without prejudice to further treatment. The investigator must withdraw any subject from the study if that subject requests to be withdrawn.

Subjects withdrawn from the study will not be replaced by the Investigator/Sponsor, regardless of the reason for withdrawal, unless they did not receive any of the investigational drugs yet.

The subject's participation in this study may be discontinued due to the following reasons:

- Request of the Sponsor or regulatory agency.
- Experiences an intolerable AE.
- Subject is unwilling or unable to continue the study or is lost to follow up.
- Subject is non-compliant with study procedures / study protocol.
- Investigator decides that withdrawal from the study is in the best interest of the subject (i.e. condition does not improve).
- Subject meets one of the exclusion criteria during the study.
- Subject needs medication not allowed in the protocol.
- Any clinically significant change in subject’s medical condition.

Subjects will be discontinued from treatment but still remain in study if any of the following occur:

- Temporary break in case of prolonged remission (>6 months)
- Significant side effects from study drug (subjects will still be followed for treatment response and safety)
- Serious or unexpected AEs (whether considered related to investigational products or not)
- Positive pregnancy test
- Serious intercurrent illness

## Handling of Withdrawals

If a subject is withdrawn from the study or fails to return either at his or her request or at the investigator’s discretion, every effort should be made to determine the reason. This information will be recorded on the subject’s case report form (CRF). All subjects who withdraw from the study prematurely, regardless of cause, should undergo all early termination assessments (see Section 5.5). It is vital to obtain follow‑up data for any subject withdrawn because of an AE or abnormal laboratory test finding. In any case, every effort must be made to undertake safety follow‑up procedures.

## Sponsor's Termination of Study

The Sponsor reserves the right to discontinue the study at any time for any reason in a time frame that is compatible with the subjects’ well-being.

# Study Procedures

## Study Procedures Chart

A schedule of events for this study is shown in Appendix A. No protocol‑related procedures, including the cessation of prohibited concomitant medications should be performed before subjects provide written informed consent. Study‑related events and activities including specific instructions, procedures, concomitant medications, dispensing of study drugs, and descriptions of AEs should be recorded in the appropriate source documents and CRF.

## Screening Visit (Day -14)

The purpose and procedures of the study will be fully explained to participating subjects. Those wishing to enroll in the study will sign a written informed consent prior to initiating any study related evaluations or procedures.

The following should be done at the screening visit, up to 14 days before initiation of treatment (Day 1):

- Review and sign Informed Consent.
- Confirm the diagnosis of metastatic gastric cancer.
- Review Inclusion and Exclusion Criteria (including NYHA cardiac classification).
- Obtain medical history and demographic data.
- Assess ECOG PS.
- Record concomitant medications and discontinue prohibited medication.
- Conduct physical examination (including vital signs, height and weight measurements).
- Conduct laboratory assessments (serum β-HCG pregnancy test for women, CBC, blood chemistry, CCT, serum electrolytes, tumor markers [CEA, CA-19.9, CA-125], coagulation markers and urinalysis).
- Conduct tumor assessment using a chest, abdominal, and pelvic CT scan.

If the time period from screening to baseline exceeds 14 days, laboratory assessment (pregnancy test, if applicable, CBC, CCT, blood chemistry, and urinalysis), vital signs and ECOG PS will be repeated.

## Baseline Visit (Day 1)

At this visit, eligible subjects will be enrolled into the study and receive study drug.

The following assessments and procedures will be done at the baseline visit:

- Review Inclusion and Exclusion Criteria and verify eligibility on the CRF.
- Assign subject number and enroll the subject.
- Conduct physical examination..
- Record vital signs (before and after each treatment and after the termination of bevacizumab infusion).
- Record concomitant medications, if any.
- Administer IV infusion of docetaxel, cisplatin, and bevacizumab.
- Dispense capecitabine.
- Record AEs, if any.

If the time period from screening to baseline exceeds 14 days, laboratory assessment (pregnancy test, if applicable, CBC, CCT, serum electrolytes, blood chemistry, urinalysis and a 24-hour urine collection), and ECOG PS will be repeated.

## Treatment phase (until toxicity, disease progression or withdrawal of consent)

The following procedures will be done at all treatment period visits (Days 1 and 8 of each treatment cycle):

- Conduct physical examination (by a physician on Day 1 of each cycle).
- Record vital signs (before and after each treatment and after the termination of bevacizumab infusion).
- Assess ECOG PS.
- Record AEs, if any.
- Conduct toxicity evaluation
- Assess treatment compliance.
- Record concomitant medication, if any.
- Conduct laboratory assessments (CBC and serum electrolytes).
- Administer IV infusion of docetaxel and cisplatin.

In addition, the following will be conducted at the beginning of each treatment cycle (Day 1):

- Dispense capeticabine
- Perform blood chemistry, tumor markers (only those elevated at baseline) and urinalysis.

Radiological tumor assessment will be done approximately every nine (9) weeks.

## Early Discontinuation Study Visit

If a subject discontinued prematurely from the study for the reasons specified in Section 4.4, the following procedures will be conducted at the Early Discontinuation Study Visit:

- Conduct physical examination.
- Record vital signs.
- Record concomitant medications.
- Record AEs.
- Conduct radiological tumor assessment (if more than 4 weeks from last assessment).
- Assess ECOG PS.
- Laboratory tests (pregnancy test, CBC, blood chemistry, tumor markers, urinalysis).
- Record survival.

## Post-Treatment Follow-Up Period (From toxicity, disease progression or withdrawal to death)

Subjects will be followed up every two (2) months from the last treatment visit. The window visits will be ±7 days.

Until progression in case of treatment discontinuation due to toxicity or patient's refusal), the following procedures will be conducted at the follow-up visits:

- Conduct physical examination.
- Record concomitant medications, if any.
- Record AEs.
- Assess ECOG PS.
- Conduct laboratory assessments including CBC, and blood chemistry.
- Conduct chest, abdominal, or pelvic CT scan every two (2) months.

This follow-up schedule will be conducted also in patients having a temporary break in their treatment ("drug holiday").

Following progression, follow-up evaluations will focus on collecting information on further anti-tumor therapies and OS time. This follow-up may be performed via phone calls to the subject, her/his family or local doctor, and will not include any specific procedures.

## Treatment Compliance

Study drug accountability will be performed immediately after study drug infusion is completed and upon returning empty or used capecitabine packages at the beginning of each cycle.

## Protocol Revisions and/or Deviations

With the exception of emergency situations, no changes or deviations in the conduct of this protocol will be permitted without the prior documented approval of the Sponsor and the Study Medical Monitor. The Institutional Review Board (IRB) that granted original approval for the study must be notified of all changes in the protocol, and must provide documented approval of any change or deviation that may increase risk to the subject, and/or that may adversely affect the rights of the subject or validity of the investigation.

In the event of an emergency, the investigator will institute any medical procedures deemed appropriate. However, all such procedures must be promptly reported to the Study Medical Monitor, and the IRB.

# Investigational Product

## Identity of Investigational Product

Roche Pharmaceuticals (Israel) Ltd. will package, code and supply capecitabine (Xeloda^TM^) and bevacizumab (Avastin^TM^) to the participating center. Sanofi-Aventis will package, code and supply docetaxel (Taxatore^TM^) to the participating center. Cisplatin will be packaged, coded and supplied by the pharmacy of the participating medical center. A sufficient supply of each study drug will be provided to the clinical site for the completion of the trial.

Xeloda^TM^ (capecitabine) is a white to off-white crystalline powder with an aqueous solubility of 26 mg/mL at 20ºC. It is supplied as biconvex, oblong film-coated tablets for oral administration. Tablets are available in two dosage strengths containing 150 mg capecitabine (light peach-colored tablet) or 500 mg capecitabine (peach-colored tablet). In the current study, only 500 mg tablets will be used. The inactive ingredients in capecitabine include: anhydrous lactose, croscarmellose sodium, hydroxypropyl methylcellulose, microcrystalline cellulose, magnesium stearate and purified water. The peach or light peach film coating contains hydroxypropyl methylcellulose, talc, titanium dioxide, and synthetic yellow and red iron oxides.

Avastin^TM^ (Bevacizumab) is a clear to slightly opalescent, colorless to pale brown, sterile, pH 6.2 solution for intravenous (IV) infusion. Avastin is supplied in 100 mg and 400 mg preservative-free, single-use vials to deliver 4 mL or 16 mL of Avastin (25 mg/mL). The 100 mg product is formulated in 240 mg α,α-trehalose dihydrate, 23.2 mg sodium phosphate (monobasic, monohydrate), 4.8 mg sodium phosphate (dibasic, anhydrous), 1.6 mg polysorbate 20, and Water for Injection, USP. The 400 mg product is formulated in 960 mg α,α-trehalose dihydrate, 92.8 mg sodium phosphate (monobasic, monohydrate), 19.2 mg sodium phosphate (dibasic, anhydrous), 6.4 mg polysorbate 20, and Water for Injection, USP.

Taxotere^TM^ (docetaxel) Injection Concentrate is a clear yellow to brownish-yellow viscous solution. It is sterile, non-pyrogenic, and is available in single-dose vials containing 20 mg (0.5 mL) or 80 mg (2 mL) docetaxel (anhydrous). Each mL contains 40 mg docetaxel (anhydrous) and 1040 mg polysorbate 80. Taxatore^TM^ Injection Concentrate requires dilution prior to use. A sterile, non-pyrogenic, single-dose diluent is supplied for that purpose. The diluent contains 13% ethanol in water for injection, and is supplied in vials.

Cisplatin (CDDP, Platinol®) is available as 10 mg and 50 mg amber vials of dry powder which are reconstituted with 10 ml and 50 ml of sterile water for Injection USP, respectively. Cisplatin is also available as a 1mg/ml solution in 50 and 100 mg vials.

## Study Drug Administration

**Part A – Phase I Study**

Subjects will be treated with the investigational regimen using escalating doses of docetaxel and cisplatin (Appendix B); the doses of capecitabine and bevacizumab will not be escalated. Patients will receive 14 days of treatment in each cycle unless they experience a DLT. Treatment will be continued until toxicity or progression or withdrawal due to any reason. Temporary discontinuation of treatment ("drug holiday"), until disease progression, will be allowed after a minimum of six months of treatment.

Treatment will be given in an outpatient setting. Patients must receive premedication with antiemetics (see section 6.10.3) and appropriate hydration for cisplatin administration.

The initial dose level for the first cohort will include docetaxel 30 mg/m^2^, cisplatin 30 mg/m^2^, two infusions per 3-week cycle on Day 1 and Day 8, and bevacizumab 7.5 mg/kg on Day 1 of each cycle. Capecitabine, 1,600 mg/m^2^/d PO, divided into two daily doses, will be dispensed at the beginning of each cycle and will be taken daily for 14 consecutive days, followed by one week rest.

On day 1 of each cycle, the sequence of drug administration will be as follows: bevacizumab will be administered first, as a 10 minute infusion, followed by a 1 hour infusion of docetaxel in 150cc normal saline (NS) and then by a 1 hour infusion of cisplatin in 500cc NS. Cisplatin administration will be preceded by an infusion of 1,000cc NS over 1 hour and followed by a 500cc NS infusion over 20-30 minutes. On that day, the morning dose of capecitabine will be taken at the time of premedication (prior to all other agents).

On day 8 of each cycle, the same sequence will take place but no bevacizumab will be given. Sequence of drug administration per cycle is described in Table 6-1. Dose modifications for study drug related toxicity is described in section 6.3, and Appendix E.

**Part B – Phase II study**

Once the recommended dose is determined, the phase II trial will be initiated.

Treatment will be given in an outpatient setting. Subjects will receive a combination therapy of cisplatin 30-35 mg/m^2^, and docetaxel 30-35 mg/m^2^, two infusions per 3-week cycle on Day 1 and Day 8, and bevacizumab 7.5 mg/kg on Day 1 of each cycle. Capecitabine, 1,600 mg/m^2^/d PO, divided into two daily doses, will be dispensed by study nurse at the beginning of each cycle and will be taken daily for 14 consecutive days, followed by one week rest. The sequence of drug administration per cycle and the antiemetic (see section 6.10.3) and hydration protocols will be identical to those used in the phase I part of the trial (see above).

Cisplatin, docetaxel, and bevacizumab will be prepared by the pharmacist and administered by the study nurse.

Dose modifications for study drug related toxicity is described in section 6.3 and Appendix E. Treatment will be continued until toxicity or progression or withdrawal due to any reason. Temporary discontinuation of treatment ("drug holiday"), until disease progression, will be allowed after a minimum of six months of treatment.

Table ‎6‑2 Sequence of Drug Administration per Cycle*

| **Study Drug** | **Day in cycle** | | | | | | | | | | | | | | | | | | | | |
| --- | --- | --- | --- | --- | --- | --- | --- | --- | --- | --- | --- | --- | --- | --- | --- | --- | --- | --- | --- | --- | --- |
|  | **1** | **2** | **3** | **4** | **5** | **6** | **7** | **8** | **9** | **10** | **11** | **12** | **13** | **14** | **15** | **16** | **17** | **18** | **19** | **20** | **21** |
| **Capecitabine** | x | x | x | x | x | x | x | x | x | x | x | x | x | x |  |  |  |  |  |  |  |
| **Bevacizumab** | x |  |  |  |  |  |  |  |  |  |  |  |  |  |  |  |  |  |  |  |  |
| **Docetaxel** | x |  |  |  |  |  |  | x |  |  |  |  |  |  |  |  |  |  |  |  |  |
| **Cisplatin** | x |  |  |  |  |  |  | x |  |  |  |  |  |  |  |  |  |  |  |  |  |

* Drugs are listed in their order of administration.

### Parameters for treatment on Day 1 of the chemotherapy cycle

Treatment should be administered after approving the following parameters:

ANC > 1,500/dl

Platelets > 100,000/dl

Serum creatinine < 1.5 mg/dl

*If the serum creatinine is >1.5 but < 2.0 mg/dl dose of cisplatin and capecitabine should be modified as outlined in Table ‎6-4 (for all other cycles than cycle 1).

### Parameters for treatment on all OTHER days (except Day 1)

ANC >1,000/mm3

platelets > 75,000/mm3

Serum creatinine < 1.5 mg/dl

*If the serum creatinine is >1.5 but < 2.0 mg/dl dose of cisplatin and capecitabine should be modified as outlined in Table ‎6-5.

## Dose Modifications for Toxicities

The dose of docetaxel, cisplatin and capecitabine will be modified on the basis of the most severe toxicity observed since the last treatment and unresolved toxicities, including abnormal laboratory results, on days 1 and 8 of each cycle, according to the criteria for dose adjustment presented in this section. The grade of toxicity should be defined as that occurring despite maximal medical management (i.e. optimal anti-emetics or intensive loperamide for diarrhea). If multiple toxicities are seen, dose modifications should be based on the most severe toxicity noted. Toxicities will be graded according to the NCI-CTCAE v 3.0. There is no dose adjustment for bevacizumab: the drug will be given in a full dose, withheld or permanently discontinued (see Section 8).

Docetaxel, cisplatin and capecitabine will not be concurrently withheld if bevacizumab is withheld. Likewise, if docetaxel, cisplatin, or capecitabine are withheld, bevacizumab will not be concurrently withheld, unless required by parameters described in Section 8. With the exception of peripheral neuropathy, nephrotoxicity and ototoxicity, in which only cisplatin will be withheld, and hand and foot syndrome, in which only capecitabine will be withheld (see below), in all other circumstances withholding one agent implies withholding all other chemotherapeutic agents as well.

In the phase I part, the starting dose for all drugs will be the dose level at which the patient is assigned to, starting at dose level 0 in the first cohort. In the phase II part, the starting dose for all drugs will be the MTD, i.e. the recommended phase II dose. While the dose levels described in Appendix B will serve for the dose finding schema in the phase I part, the dose levels per drug described below will serve for the toxicity-related dose modifications for each drug, in both parts of the study. The dose levels are relative to the starting dose the patient was receiving. One should distinguish between the planned dose modifications as per the dose escalation schema described in Appendix B and unplanned dose modifications following toxicity described here.

If chemotherapy is withheld due to blood test abnormalities, these blood tests will be repeated twice a week until chemotherapy can be resumed. A toxicity-related treatment delay of up to two (2) weeks is allowed: if chemotherapy can not be resumed after a delay of 2 weeks, the patient will be taken off study. Continuing the study regimen in these circumstances will be allowed if, in the judgment of the treating physician, this will be in the benefit of the patient. A treatment-related toxicity of more than one (1) week is allowed but will require a dose attenuation of one (1) dose level of all three drugs aside of bevacizumab.

Once a dose modification for toxicity since the last treatment has been performed, the new dose will not be increased. On the other hand, dose modification of cisplatin for mild elevation of creatinine on the day of treatment, may be reversible.

Table ‎6‑3 Docetaxel/CDDP/capecitabine Dose Levels

| **Docetaxel** | 20 mg/m^2^ | 25 mg/m^2^ | 30 mg/m^2^ | 35 mg/m^2^ |
| --- | --- | --- | --- | --- |
| **CDDP** | 20 mg/m^2^ | 25 mg/m^2^ | 30 mg/m^2^ | 35 mg/m^2^ |
| **Capecitabine** | 950 mg/m^2^ | 1150 mg/m^2^ | 1350 mg/m^2^ | 1600 mg/m^2^ |

Table ‎6‑4 Dose Modifications according to Laboratory Results on Day 1 of the cycle (except cycle 1)

|  | **Docetaxel** | **CDDP** | **Capecitabine** |
| --- | --- | --- | --- |
| **ANC** |  |  |  |
| > 1500/dl | Maintain dose level | Maintain dose level | Maintain dose level |
| 1000-1499/dl | Hold | Hold | Hold |
| <1000/dl | Hold | Hold | Hold |
| **PLT** |  |  |  |
| > 100,000/dl | Maintain dose level | Maintain dose level | Maintain dose level |
| 75,000-99,000/dl | Hold | Hold | Hold |
| <75,000/dl | Hold | Hold | Hold |
| **Creatinine** |  |  |  |
| < 1.5 mg/dl | Maintain dose level | Maintain dose level | Maintain dose level |
| 1.5-2.0 mg/dl | Maintain dose level | Decrease 2 dose levels | Decrease 1 dose level |
| > 2.0 mg/dl | Maintain dose level | Hold | Hold |

Table ‎6‑5 Dose Modifications according to Laboratory Results on all OTHER days (including Day 8)

|  | **Docetaxel** | **CDDP** | **Capecitabine** |
| --- | --- | --- | --- |
| **ANC** |  |  |  |
| > 1000/dl | Maintain dose level | Maintain dose level | Maintain dose level |
| <1000/dl | Hold | Hold | Hold |
| **PLT** |  |  |  |
| > 75,000/dl | Maintain dose level | Maintain dose level | Maintain dose level |
| <75,000/dl | Hold | Hold | Hold |
| **Creatinine** |  |  |  |
| < 1.5 mg/dl | Maintain dose level | Maintain dose level | Maintain dose level |
| 1.5-2.0 mg/dl | Maintain dose level | Decrease 2 dose levels | Decrease 1 dose level |
| > 2.0 mg/dl | Maintain dose level | Hold | Hold |

Table ‎6‑6 Dose Modifications for Hematological Toxicity (since the last treatment)

|  | **Docetaxel** | **CDDP** | **Capecitabine** |
| --- | --- | --- | --- |
| **ANC (nadir)** |  |  |  |
| > 500/dl | Maintain dose level | Maintain dose level | Maintain dose level |
| < 500/dl | Decrease 2 dose levels | Decrease 1 dose level | Decrease 1 dose level |
| **Neutrop. fever** | Decrease 2 dose levels | Decrease 1 dose level | Decrease 1 dose level |
| **PLT (nadir)** |  |  |  |
| > 25,000/dl | Maintain dose level | Maintain dose level | Maintain dose level |
| < 25,000/dl | Decrease 2 dose levels | Decrease 2 dose levels | Decrease 1 dose level |
| **Bleeding** | Decrease 2 dose levels | Decrease 2 dose levels | Decrease 1 dose level |

Table ‎6‑7 Dose Modifications for Non-Hematological Toxicity (since the last treatment)^1^

|  | **Docetaxel** | **CDDP** | **Capecitabine** |
| --- | --- | --- | --- |
| **Nausea/Vomiting** |  |  |  |
| Grade < 2 | Hold until grade < 2; maintain dose level | Hold until grade < 2; decrease 1 dose level | Hold until grade < 2; maintain dose level |
| Grade > 3 | Hold until grade < 2; decrease 1 dose level | Hold until grade < 2; decrease 1 dose level | Hold until grade < 2; decrease 1 dose level |
| **Mucositis/Diarrhea** |  |  |  |
| Grade < 2 | Hold until grade < 2; maintain dose level | Hold until grade < 2; maintain dose level | Hold until grade < 2; maintain dose level |
| Grade 3 | Hold until grade < 2; decrease 1 dose level | Hold until grade < 2; decrease 1 dose level | Hold until grade < 2; decrease 1 dose level |
| Grade 4 | Hold until grade < 2; decrease 2 dose levels | Hold until grade < 2; decrease 1 dose level | Hold until grade < 2; decrease 2 dose levels |
| **Hand and Foot** |  |  |  |
| Grade < 2 | Maintain dose level | Maintain dose level | Hold until grade < 2; maintain dose level |
| Grade 3 | Maintain dose level | Maintain dose level | Hold until grade < 2; decrease 1 dose level |
| **Neuropathy** |  |  |  |
| Grade 1 | Maintain dose level | Maintain dose level | Maintain dose level |
| Grade 2 | Maintain dose level | Decrease 1 dose level | Maintain dose level |
| Grade > 3 | Hold until grade < 2; decrease 1 dose level | Hold until grade < 2; decrease 1 dose level | Maintain dose level |
| **Ototoxicity** |  |  |  |
| Grade < 2 | Maintain dose level | Maintain dose level | Maintain dose level |
| Grade > 3 | Maintain dose level | Hold until grade < 2; decrease 1 dose level | Maintain dose level |

^1^ Unless otherwise specified in the table, treatment with docetaxel, CDDP and capecitabine will be withheld until all non-hematological toxicities resolve to grade < 2. Similarly, unless otherwise specified in the table, the dose of the three drugs will be decreased by one dose level for any grade > 3 non-hematological toxicities.

## Manufacturing

Capecitabine (Xeloda^TM^) and Bevacizumab (Avastin^TM^) are manufactured by Roche.

Docetaxel (Taxatore^TM^) is manufactured by Sanofi-Aventis.

Cisplatin (Platinol®) is manufactured by Bristol-Myers Squibb.

## Packaging and Labeling

Cisplatin will be labeled as customary for regular oncology clinic use; it will not be labeled for clinical trial use.

Taxotere^TM^, Xeloda^TM^, and Avastin^TM^ will be labeled for clinical trial in English and Hebrew.

## Distribution and Shipment

Each shipment of Xeloda^TM^ and Avastin^TM^ study drug supplies, sent by Roche, and Taxotere^TM^ study drug supplies, sent by Sanofi-Aventis, will contain a shipment form describing the content of shipment. This form will assist in maintaining current and accurate inventory records. When a shipment is received, the investigator and/or study coordinator and/or pharmacist will verify its content and acknowledge receipt of the study drug supply by signing the shipment form and faxing it back to Roche or Sanofi-Aventis attention.

If, upon arrival at the investigational site, the study drug supplies appear to be damaged, or missing, the Sponsor should be contacted immediately.

Cisplatin will be purchased by the hospital pharmacy from the appropriate vendor.

## Storage, Dispensing and Return of the Investigational Product

Study drug supplies must be kept according to their storage conditions appearing on the labels, in a secure, limited access and temperature-controlled storage area.

Only authorized personnel will have access to the study drug supplies.

The study site personnel at each site will be responsible for maintaining proper storage and handling of the study products. The study drug supplies must be kept under the prescribed conditions.

Only personnel under the supervision of either the investigator or the local pharmacist are authorized to dispense and administer study drug. Cisplatin, docetaxel, and bevacizumab will be prepared by the pharmacist and administered IV by a study nurse to subjects participating in the clinical trial. Capecitabine will be dispensed to participating subjects at the beginning of each cycle.

## Accountability and Compliance of Investigational Product

Subject compliance with cisplatin, docetaxel, and bevacizumab dosing regimen will be assessed by study staff administrating the study drug. Subjects will be request to return empty or used capecitabine packages at the beginning of each cycle.

A drug accountability log will be used at the study center to keep accurate records of study drug inventory at the center (date & quantity received by the Roche/Sanofi-Aventis, administered to the subjects, return to the Roche/Sanofi-Aventis or alternative disposition of unused products).

Drug accountability logs record quantities received from Roche/Sanofi-Aventis and quantities dispensed to patients, including lot number, date dispensed, patient identifier number, patient initials, protocol number, dose, quantity returned, batch number, expiry date and the initials of the person dispensing the medication has to be maintained per institutional standards.

The investigator or designated staff member will be responsible for maintaining accurate records of the quantity and dates of all study drug supplies received, administered, and returned. The quantity of study drug lost, missing, destroyed, etc. must also be accounted for and documented. At the end of the study reconciling the delivery records with those of usage and returned stocks must be possible. Accounts must be given on any discrepancies.

Government regulations require that all study drug materials not used in clinical trials be returned to the Supplier (Roche/Sanofi-Aventis) before or at the completion of the study. The investigator will return the designated copies of the completed dispensing and inventory record as indicated on the form.

## Storage of Investigational Product

All study drug sent to the study center must be stored under the conditions specified in the package insert (package inserts are available separately at the participating center) and in a secure area accessible only to the investigator and designated site personnel. All study drugs should be stored and inventoried according to applicable government regulations and study procedures.

## Prior and Concomitant Therapy

### General guidelines

All prior treatments received by the subject within 30 days of the initial Screening visit will be recorded on the subject’s CRF including the treatment's name and the start and stop dates.

Any medications (including prescription, over-the-counter, herbal supplements and health store products) to be taken during the study must be approved by the investigator.

All approved concomitant medications taken by the subject must be recorded on the CRF, along with the start and stop dates as well as daily dose.

### Anticancer or experimental therapy

No other concurrent chemotherapy of any kind is permitted while the subject is receiving study treatment.

### Anti-emetics

A standard anti-emetic premedication protocol for high emetogenic chemotherapy should be used. The IV component of the premedication regimen will start prior to the administration of any agent, including bevacizumab, on Days 1 and 8 of each cycle.

Granisetron 3mg IV or Ondansetron 8mg + Dexamethasone 12mg + Cimetidine 400mg PO will be administered on treatment days (Days 1 and 8). Aprepitant 125mg PO will be given on each treatment day, followed by two single daily doses of 80mg on the subsequent days. In case of substantial nausea/vomiting, single daily doses of Dexamethasone 8mg PO may be added on days 2,3 and 9,10 in subsequent cycles (with Cimetidine 400 mg PO on those days).

Granisetron 1mg or Ondansetron 8mg PO will be taken on the evening of each treatment day. Granisetron 1mg, or Ondansetron 8mg PO will be taken twice daily two days following each treatment (three times daily in subsequent cycles, if significant nauseas/vomiting occur).

PRN antiemetics: Metoclopramide 10 mg PO every six hours PRN nausea/vomiting, and/or Lorazepam 1-2 mg PO every 4 - 6 hours PRN nausea or anticipatory anxiety.

### Hematopoetic Growth Factors

Colony-Stimulating Factor (G-CSF, GM-CSF):

Initial prophylactic use of colony-stimulating factors (G-CSF, GM-CSF, Pegfilgrastim) is not allowed. However, prophylactic administration of G-CSF in a patient who is experiencing difficulty with neutropenia (eg. protracted neutropenia), or therapeutic use in patients with serious neutropenic complications such as tissue infection, sepsis syndrome, fungal infection, etc., may be considered at the investigator's discretion. It should be emphasized however that **administration of colony-stimulating factors concomitantly (on the same day) with chemotherapy, including capecitabine, is not allowed**.

Erythropoetin:

Use of erythropoetin alfa is permitted at the discretion of the treating physician.

### Prohibited concomitant medication

The following medications are not permitted during the study and may lead to withdrawal of the subject from the study:

- Other chemotherapy of any kind
- Drugs that induce, inhibit, or are metabolized by cytochrome P450 3A4, such as cyclosporine, terfenadine, ketoconazole, erythromycin, and troleandomycin
- Allopurinol
- Sorivudine or its chemically related analogues, such as brivudine (an antiviral drug contraindicated for capecitabine)

The dose of phenytoin and the dose of Coumadin® may need to be reduced when either drug is administered concomitantly with Xeloda^TM^.

See package insert of Taxotere^TM^ (docetaxel), Xeloda^TM^ (capecitabine), and Avastin^TM^ (bevacizumab) for additional information on prohibited concomitant medications and contraindications (package inserts will be available at the participating centers).

# Assessment Procedures

## Safety Assessments and Endpoints

Safety assessments will be based on changes from Baseline of clinical AEs reported by the subject or observed by the investigator, concomitant medication use, treatment compliance (e.g. dropouts due to AEs), vital sigh, physical examination, and laboratory assessments (pregnancy test, CBC, blood chemistry, and urinalysis).

### Adverse events

Adverse events will be reported and graded in accordance with the National Cancer Institute Common Terminology Criteria for Adverse Events (CTCAE) version 3 (see section 8.0).

AEs for cisplatin may include anorexia, nausea, vomiting, renal toxicity (with an elevation of BUN and creatinine, as well as tubular damage which is usually transient), ototoxicity (hearing loss, initially in the high-frequency range, and tinnitus), hyperuricemia, myelosupression (most commonly mild-moderate anemia), peripheral neuropathy, loss of taste, allergic reactions, seizures, ocular toxicities, rare cardiac abnormalities, or possible acute myeloid leukemia.

Dose-limiting toxicities with weekly docetaxel administration include fatigue and asthenia. Myelosuppression is relatively uncommon with the weekly dosing regimen. Patients with underlying hepatic dysfunction may have more severe myelosuppression. Hypersensitivity reactions are relatively uncommon, and tend to occur during the first several doses of docetaxel. There can be a significant range of hypersensitivity reactions, from mild reactions (manifested as mild flushing, drug fever, chest tightness, dyspnea, or chills) to severe reactions (manifested as angioedema, hypotension, bronchospasm, and generalized rash). Hypersensitivity reactions are more common and severe in patients with hepatic dysfunction.

Other potential toxicities observed with docetaxel include fluid retention, skin eruptions (erythema with desquamation, nail changes), alopecia, fatigue, peripheral neuropathy, gastrointestinal toxicities (low incidence of nausea, vomiting, diarrhea; and mucositis), and hyperlacrimation. Caution with administration is advised given the potential for extravasation injury with docetaxel.

The most commonly expected (>10%) AEs for docetaxel in combination with cisplatin include anemia, neutropenia, thrombocytopenia, nausea, vomiting, diarrhea, constipation, stomatitis, fever, lethargy, and alopecia.

The most common expected AEs for capecitabine include gastrointestinal disorders, especially diarrhoea, nausea, vomiting, stomatitis, and hand-foot syndrome (palmar-plantar erythrodysesthesia). Anorexia, fatigue, asthenia, and neutropenia are also common (>10%).

When capecitabine is administered in combination with cisplatin, leucopenia, and anemia are also expected.

Common AEs (>10%) of capecitabine in combination with docetaxel are neutropeic fever (grade 3-4), decreased appetite, taste disturbance, increased lacrimation, lower limb edema, sore throat, constipation, dyspepsia, alopecia, nail disorder, myalgia, arthralgia, pyrexia, and weakness.

The most frequently observed AEs for bevacizumab are Leucopenia, Thrombocytopenia, Neutropenia, Peripheral sensory neuropathy, hypertension, fatigue or asthenia, diarrhea and abdominal pain. Other common AEs include bleeding and proteinuria. Rare but severe AEs of bevacizumab include thromboembolic events (arterial and venous) and perforation of gastrointestinal viscera. Additional AEs of special interest (AEs that were observed across clinical trials in patients who have received bevacizumab in combination with different chemotherapy regimens) are: congestive heart failure, wound healing complication, fistula, reversible posterior, leukoencephalopathy syndrome,

Dose modifications for study drugs related toxicities are described in section 6.3 and Appendix E.

For additional information on adverse events see section 4.8 in package inserts (package inserts will be available at the participating center) of Taxotere^TM^ (pages 24-31, 44-45 in package insert October 2006), Xeloda^TM^ (pages 9-16 in package insert July 2007), and Avastin^TM^ (pages 6-12 in package insert September 2008).

### Concomitant medication use

Recording of concomitant medication use will be conducted at each study visit.

### Treatment compliance

Cisplatin, docetaxel, and bevacizumab drug accountability will be performed immediately after infusion is completed.

Capecitabine drug accountability will be performed after the subject returns used study drug packages. Subjects will be requested to return used packages at the beginning of each cycle.

### Vital signs

Vital signs measurements will be obtained at all study visits, including early termination, and will include temperature, blood pressure and heart rate after the subject has sat quietly for at least 5 minutes. On treatment days (days 1 and 8 of each treatment cycle), vitals signs will be measured at the initiation of bevacizumab infusion.

### Physical Examination

A complete physical examination will be performed at Screening, treatment, early termination, and follow up visits. The physical examination includes appearance, eyes, ears, nose, head, throat, neck, chest, lungs, heart, abdomen, extremities, skin, and musculoskeletal system. Height and weight will be measured at the Screening visit with the subjects wearing normal indoor clothes but without shoes.

### Laboratory Assessments

All routine clinical laboratory assessments will be performed by local laboratory of the participating center. The clinical laboratory evaluation will be performed at Screening/Baseline, and at predetermined study visits or early termination visit.

The laboratory evaluations will include:

1. Hematology: hemoglobin, hematocrit, RBC count, WBC count with differential, and platelet count. This will be done at each study visit.
2. Blood chemistries: sodium, potassium, glucose, cholesterol, calcium, magnesium, creatinine, phosphorus, BUN, uric acid, total bilirubin, total protein, AST, ALT, GGT, and alkaline phosphatase levels. This will be done at the beginning of each treatment cycle. Electrolytes test ("small chemistry"), including sodium, potassium, chloride, magnesium, creatinine, and BUN, will be done at each study visit.
3. Creatinine clearance will be tested only at screening and may be repeated at baseline.
4. A 24-hour urine collection will be done at screening and may be repeated at baseline.
5. Pregnancy Tests: a serum β-HCG or urine pregnancy test for women of childbearing potential will be performed at Screening and at early termination visit.
6. Coagulation Markers: PT/PTT/Fibrinogen will be done at Baseline visit.

All laboratory tests with values that became abnormal after drug administration will be repeated as clinically indicated until the values return to normal, or until the etiology has been determined and the condition considered stable. Abnormal laboratory results that are considered to be clinically important by the investigator will be reported as an AE in the CRF. A laboratory abnormality will not be considered an AE unless:

- Intervention is required.
- Changes in dose are required (decrease, discontinued, interrupted).
- Other treatment/therapy is required.
- Associated with other diagnoses.

## Efficacy Assessment

### Tumor Assessment

Measurements of tumor parameters will be done at the screening visit, at treatment period every nine (9) weeks, and during follow-up period (every three months). Tumor response will be evaluated according to Response Evaluation Criteria in Solid Tumors (RECIST) guidelines [41] (see Appendix D). One (1) to five (5) measurable lesion(s) will be identified during screening CT. The sum of the largest perpendicular diameter of all target lesions will be used to measure tumor response. A complete response (CR) is defined as disappearance of all target lesions, all non-target lesions, and return to normal levels of CEA levels (if evaluable). A partial response (PR) is defined as at least a 30% decrease in the sum of the longest diameter of target lesions. Stable disease (SD) is neither sufficient shrinkage to qualify for partial response nor sufficient increase to qualify for progressive disease. Progressive disease (PD) is at least a 20% increase in the sum of the longest diameters of target lesions or the appearance of one or more new tumor lesions, and/or unequivocal progression of existing non-measurable lesions.

### ECOG Performance Scale

The Eastern Cooperative Oncology Group (ECOG) Performance Status will be used to assess performance status by the same study staff (Appendix C). The performance status will be assessed at every visit, including early termination and unscheduled visit.

# Safety and Pharmacovigilance

## Adverse Event

An adverse event (AE) is any adverse change from the subject baseline condition, whether or not considered investigational product related. This includes any subjective signs, symptoms or diagnosis, clinical significant deviation from baseline laboratory values or vital signs, or worsening (more severe, more frequent or increased in duration during the investigational product treatment) of the concomitant disease present at baseline visit (after initiation of investigational product treatment). Stable chronic conditions that are present prior to study entry and do not worsen during the study will not be considered AEs. Disease-related adverse events will not be considered AEs only if they worsen beyond what would be expected in the normal progression of the disease. In all cases, the etiology should, as much as possible, be identified and the Sponsor notified.

An abnormal result of diagnostic procedures including abnormal laboratory or vital sign findings will be considered an AE if it:

- Results in subject’s withdrawal by the investigator
- Is associated with clinical signs or symptoms
- Is considered by the physician to be of clinical significance

Adverse events reported by the subject or observed by the investigator will be individually listed on an adverse event form in the CRF as follows: the specific event or condition, whether the event was present pre-study, the dates and times of occurrence, duration, severity, relationship to study medication, specific countermeasures, and outcome.

Adverse event severity (Table ‎8-8) will be recorded and graded according to the National Cancer Institute (NCI) Common Terminology Criteria for Adverse Events (CTCAE), version 3.0 (August 2006) [42]. The CTC manual can be found at the following website <http://ctep.cancer.gov/reporting/ctc.html>.

Table ‎8‑8 Severity of Adverse Events According to CTCAE

| **Grade** | **Description** |
| --- | --- |
| 0 | No AE or within normal limits |
| 1 | Mild AE |
| 2 | Moderate AE |
| 3 | Severe AE |
| 4 | Life-threatening or disabling AE |
| 5 | Death related to AE |

The following definitions should be used for toxicities that are not defined in the CTCAE:

- Mild (Grade 1): the AE is noticeable to the subject but does not interfere with routine activity;
- Moderate (Grade 2): the AE interferes with routine activity but responds to symptomatic therapy or rest;
- Severe (Grade 3): the AE significantly limits the subject’s ability to perform routine activities despite symptomatic therapy;
- Life-threatening (Grade 4): the subject is at immediate risk of death.

The investigator will document his opinion of the relationship of the AE to treatment with investigational product using the criteria outlined in Table ‎8-9.

Table ‎8‑9 Relationship of Adverse Event to Treatment

| **Relationship** | **Description** |
| --- | --- |
| Unrelated | The AE *is clearly NOT related* to the study treatment. |
| Unlikely | The AE *is doubtfully related* to the study treatment. |
| Possible | The AE *may be related* to the study treatment. |
| Probable | The AE *is likely related* to the study treatment. |
| Definite | The AE *is clearly related* to the study treatment |

Outcome to Date are classified as follows:

1. Recovered – The subject has fully recovered from the adverse event with no residual effects observable.
2. Recovered with sequelae – The subject has fully recovered from the adverse event with no residual effects observable.
3. Ongoing – Adverse event is still ongoing.

## Serious Adverse Event

A **serious** adverse event (SAE) is any adverse event occurring at any dose that suggest a significant hazard or side effect, regardless of the investigator or Sponsor's opinion on the relationship to the investigational product and that results in, but may not be limited to, any of the following outcomes:

- death (regardless of the cause)
- a life-threatening adverse drug experience
- inpatient hospitalization or prolongation of existing hospitalization (any inpatient hospital admission that includes a minimum of an overnight stay in a health care facility)
- a persistent or significant disability/incapacity
- a congenital anomaly or birth defect
- Important medical events that may not result in death, be life-threatening, or require hospitalization may be **serious** when, based upon appropriate medical judgment, they may jeopardize the subject and may require medical or surgical intervention to prevent one of the outcomes listed above.

Hospitalization for elective treatment of a pre-study condition that did not worsen while on study and hospitalizations for treatment of non-adverse events (e.g. cosmetic surgery) are not considered serious adverse events.

**Significant medical events** are those which may not be immediately life-threatening, but may jeopardize the subject and may require intervention to prevent one of the other serious outcomes listed above. Examples of such events are intensive treatment in an emergency room or at home for allergic bronchospasm; blood dyscrasias or convulsions that do not result in hospitalization; resulting in an adverse event will normally be considered serious by this criterion.

Inpatient hospitalization or prolongation of existing hospitalization means that hospital inpatient admission and/or prolongation of hospital stay were required for treatment of AE, or that they occurred as a consequence of the event. Hospitalization for elective treatment of a pre-study condition that did not worsen while on study and hospitalizations for treatment of non-adverse events (e.g. cosmetic surgery or diagnostic procedure) are not considered serious adverse events.

Any new SAE that occurs after the study period and is considered to be related (possibly/probably) to the investigational product or study participation should be recorded and reported immediately

A **life-threatening** adverse drug experience is any adverse event that places the subject, in the view of the investigator, at immediate risk of death from the reaction as it occurred, i.e., it does not include a reaction that, had it occurred in a more severe form, might have caused death.

## Definition of an Unexpected Adverse Event

An **unexpected** adverse drug experience (event) is any adverse event, the specificity or severity of which is not consistent with information in the current Investigator’s brochure for an unapproved investigational product or package insert/summary of product characteristics for an approved product (package inserts are available separately at the participating center).

## Notification about Serious or Unexpected Adverse Events

The investigator must record all serious adverse and/or unexpected events, regardless of treatment or relationship to investigational product as soon as s/he in informed of the event. The investigator must also notify within 24 hours the Study Medical Monitor by phone or by fax by sending the "Notification of Serious Adverse Event" form located in the investigator site file.

Medical Monitor: Dr. Baruch Brenner Daytime Telephone: 03-9378076 Fax: 03-9231437

Within 3 working days from the time that the investigator was notified of the **serious** adverse event, the corresponding Clinical Adverse Event Form from the CRF (and copies of the medication logs and any other required documentation) will be faxed to the Study Medical Monitor.

SAEs should be reported by the Sponsor to EC/IRB according to local requirements. Subjects who have had an SAE during the treatment period must be followed clinically until all parameters (including laboratory), have either returned to normal, have stabilized or are otherwise explained.

# Statistical Analysis Plan

## Statistical Plan

This is a non-randomized Phase I/II study of a combination of weekly docetaxel and cisplatin together with capecitabine and bevacizumab in patients with advanced gastric cancer. All enrolled patients will have histologically proven metastatic or unresectable gastric adenocarcinoma. The study has two primary objectives. The primary objective of the phase I component is to define the MTD of the investigational regimen and its recommended phase II dose. The primary objective of the phase II component is to evaluate the activity of the investigational regimen in terms of RR.

The phase I part of the trial was designed using a conventional dose-escalation schema. The MTD is defined as one dose level below that at which two or more patients experienced DLT. The described study design provides a 91% chance of dose escalation if the true incidence of DLT at that dose level is 10%, a 31% chance of escalation if the true incidence is 40% and only a 3% chance of escalation if the true incidence is 70%. The activity of the new regimen will be evaluated in the phase II part of the study.

The study has 4 secondary objectives:

1. Determine the safety of the investigational regimen in terms of acute and subacute toxicity profile.
2. Determine PFS.
3. Determine OS.
4. Identify patient and tumor characteristics that are associated with response to the investigational regimen and to patient outcome.

The safety of the study regimen will be assessed as the proportion of patients developing toxicity of any grade and those developing severe toxicities (grade≥3) within 30 days following the completion of investigational treatment. The safety analysis will include all patients who will receive at least one dose of study drugs.

PFS and OS will be calculated using the Kaplan-Meier method. OS will be calculated from registration to death or to the last date the patient was known to be alive. PFS will be calculated from registration to progression or to the last date the patient was known to be progression-free. The prognostic and predictive role of various patient and tumor characteristics will be determined as a statistically significant correlation between their presence and response to therapy (predictive factors) and patient outcome (prognostic factors). P values of 0.05 or less will be considered statistically significant. All secondary analyses will be exploratory in nature since the study size does not have sufficient power to provide conclusive evidence.

## Determination of Sample Size

The phase I component of the study will include up to 15 subjects with advanced gastric cancer. There will be three (3) dose levels, with 3-6 patients per cohort.

The phase II component of the study will be conducted according to Simon’s two-stage design. In the first stage, 17 patients will be enrolled. If more than three (3) patients respond in the first stage, 20 more patients will be enrolled, for a total of 37 patients. The phase II cohort will include the patients in the last cohort of the phase I (receiving the phase II recommended dose). The investigational regimen will be recommended for further evaluation if 11 or more responses are seen out of 37 patients. This design has a 10% chance of recommending the regimen for further study if the true RR is 20% or less. This probability increases to 90% if the RR is 40% or higher.

## Subject Disposition

Reasons for individual subject premature study discontinuation and premature study drug treatment discontinuation will be tabulated by study drug dose.

## Demographic and Baseline Characteristics

The descriptive statistics of demographic and baseline characteristic data will be summarized by study drug dose and tested by a chi-square test for categorical variables and a two-sided t-test for continuous variables.

## Subject Compliance

Summary tables of subject treatment compliance rating will be generated by visit and by dose, which will be analyzed by chi-squared test.

## Concomitant Medication

Concomitant medications will be tabulated using World Health Organization (WHO) drug classifications. The number of subjects using concomitant medications will be summarized.

## Extent of Exposure

The number of subjects will be summarized by number of study drug administration and by demographic characteristics (age, gender and race).

## Safety Assessment

Descriptive statistics will be calculated for laboratory parameters, physical exam, ECOG performance scale and vital signs as well as for changes from baseline of vital signs and ECOG performance scale. Details of all pregnancies that occur, if any, will be presented.

The incidence of adverse events during the treatment period will be summarized. All adverse events will be listed together with information on onset, duration, frequency, severity, seriousness, relationship to the study drug, outcome and action taken. Frequency tables for severity and relationship to study medication will be provided by study drug dose.

Adverse events will be classified by System Organ Class and preferred term according to the Medical Dictionary for Regulatory Activities (MedDRA). The latest version available at the time of coding will be used throughout the study.

Incidence of adverse events during treatment will be compared using Fisher's exact test.

One AE may consist of several components on the data set. For example if the intensity or relationship to study drug has changed, this may be recorded separately as a distinct AE. Therefore, AE records will be considered components of a single event if the preferred terms match and the dates imply AE continuity. In the summary tables, events will be counted, rather than the individual components (that is such adverse events will be merged). In the listings, however, each AE component will be presented, with a flag to indicate continuation of a previous event.

Only treatment emergent adverse events will be included in the summary tables.

## Efficacy Assessment

All measured variables and derived parameters will be listed individually and, if appropriate, tabulated by descriptive statistics. Major efficacy parameters will be graphically presented as well. For descriptive statistics summary tables will be provided giving sample size, absolute and relative frequency of categorical variables, arithmetic mean, standard deviation, coefficient of variation (if appropriate), median, minimum and maximum, percentiles and 95% CI (Confidence Interval) for means of continuous variables.

## Interim Analysis

Interim analyses will be done within the phase I study period, following each cohort. The main purpose of the interim analyses is to evaluate the study drug safety profile. The interim analyses will be done for the primary endpoint.

To assure the safety profile of the study regimen, a predetermined interim safety analysis is planned at the time at which ten patients in the phase II study have completed at least two cycles of treatment. At this point toxicity data available from both parts of the study (the completed phase I component and the ongoing phase II component) will be analyzed and the safety of the study regimen will be assessed. If, in the judgment of the Principal Investigator (PI), the regimen is found to be associated with excessive toxicity several options exist: the study regimen may be modified, the study may be closed early, or the study will continue with an additional interim safety analysis planned.

## Deviation from Original Analysis Plan

A detailed statistical analysis plan will be developed prior to database lock. Any deviation from the detailed and the original protocol statistical plan will be described and justified in the final clinical study report.

# Ethics

## Institutional Review Board or Independent Ethics Committee

Prior to initiation of the study, the PI will submit the study protocol and amendments, sample Informed Consent Form (ICF), and any other documents that may be requested to the IRB/IEC for review and approval. The PI will request that the IRB/IEC provide written approval of the study and will keep on file records of approval of all documents pertaining to this study. The PI will not begin the study until the protocol and ICF have been approved by the IRB or IEC. The PI must agree to make any required progress reports to the IRB, as well as reports of SAEs, life‑threatening problems, or death.

## Ethical Conduct of the Study

All clinical work conducted under this protocol is subject to GCP rules. This includes an inspection by Clalit Health Services or its designee, health authority or IRB representatives at any time. The investigator must agree to the inspection of study-related records by health authority representatives and/or Clalit Health Services or its designee.

The study will be conducted in accordance with Clalit Health Services standards and the following guidelines:

- GCP: Consolidated Guideline (International Conference on Harmonization of Technical Requirements for the Registration of Pharmaceuticals for Human Use, May 1996).
- Declaration of Helsinki: Seoul, 2008 (Appendix F).
- Israeli MOH guidelines (January 2006)

## Subject Information and Consent

Prior to screening for the study each subject will be informed in detail about the study drugs to be administered, and the nature of the clinical investigation with its risks and discomforts to be expected. The basic elements of informed consent as specified by the FDA (21 CFR 50.25) and ICH-GCP will be followed. Written consent will be obtained from each subject to be involved in the clinical trial by using the IRB/IEC-approved Informed Consent Form (ICF) prior to the conduct of any study-related activity. Each subject will be given a copy of the written ICF. The subjects will also be instructed that they are free to withdraw their consent and discontinue their participation in the study at any time without prejudice. Each subject’s chart will include the signed ICF for study participation. When the study treatment is completed and the CRF has been monitored, the ICF will be kept in the investigator’s central study file for the required period of time. Regulatory authorities may check the existence of the signed ICF in this central study folder if not having done so during the study.

## Subject Insurance

The PI has an insurance policy for the total duration of the study covering the subjects and investigators in respect of the risks involved in conducting this study according to this protocol. The insurance policy will be filed in the investigator's file or can be made available to the Investigator and to the IEC/IRB upon request.

## Informing the General Practitioner

The investigator will inform the subject's primary care physician of his/her participation in the study, by sending a letter to the physician as required by Israeli authorities.

## Personal Data Protection

RMC and Clalit Health services comply with the principle of subject's right to protection against invasion of privacy. Throughout this trial, all data will be identified only by an identification number and subject initials. The data will be blinded in all data analyses. The subject must be informed and consent is required that authorized personnel of RMC and/or Clalit Health services and/or designee (Study Monitor, Auditor, etc.) and relevant Health regulatory agency will have direct access to personal medical data to assure a high quality standard of the study.

## Protocol Amendments

The protocol must be read thoroughly and the instructions must be followed exactly. Any changes in the protocol will require a formal amendment. Such amendments will be agreed upon and approved in writing by the investigator/sponsor. The IRB or IEC will be notified of all amendments to the protocol. Amendments to the protocol will not be implemented until written IRB or IEC approval has been received.

# Quality Control and Quality Assurance

RMC and Clalit Health services are maintaining a quality assurance system with written SOPs to ensure that clinical trials are conducted and data are generated, documented and reported in compliance with the protocol, GCP and applicable regulatory requirements.

## Study Monitoring

Monitoring of the study is the responsibility of the Sponsor and may be delegated to a CRO or a contract monitor. The study monitor will advise the Investigator regarding the practical conduct of the study and maintaining compliance with the protocol, GCP and all applicable regulatory requirements. Throughout the course of the study, the study monitor will oversee the conduct and the progress of the study by frequent contacts with the investigator. This will include telephone calls and on-site visits. During the on-site visits, the CRF will be reviewed for completeness with corresponding source documents. As part of the data audit, source documents will be made available for review by the study monitor. The study monitor will also perform drug accountability checks and may periodically request review of the investigator study file to ensure completeness of documentation in all respects of clinical study conduct.

Upon completion of the study, the study monitor will arrange for a final review of the study files after which the files should be secured for the appropriate time period. The investigator or appointed delegate will receive the study monitor during these on-site visits, cooperate in providing the documents for inspection, and respond to inquiries.

### Source Document

The investigator will permit study-related monitoring, audits by or on behalf of the Institution (RMC), Clalit Health Services, IEC/IRB review and regulatory inspections providing direct access to source data documents. Source documents are original records in which raw data are first recorded. These may be office/clinic/hospital records, charts, diaries, x-rays, and laboratory results, printouts, pharmacy records, care records, completed scales for each study participant. Source documents should be kept in a secure and limited access area. All source documents must be accurate, clear, unambiguous, permanent and capable of being audited. They should be made using a permanent form of recording (ink, typing, printing, optical disc etc). They should not be obscured by correcting fluid or have temporary attachments (such as removable self-stick notes). Source documents that are computer generated and stored electronically must be printed, singed and dated by the investigator.

Source data for subjects registered to the study should indicate date informed consent was signed, participation in clinical protocol number and title, treatment number, evidence that inclusion/exclusion criteria have been met.

There will be 100% source data verification for this study.

### Case Report Form (CRF)

The development of the CRF will be the responsibility of Dr. Baruch Brenner or designee. The investigator will be responsible for the timeliness, completeness, and accuracy of the information on the CRF. All entries must be legibly recorded in black ink. The entry to be corrected is to be crossed out with a single line so that the original entry remains legible. The correction then has to be made right next to the entry and confirmed by date and initials of the person making the correction. Corrections that cannot be made in this fashion have to be explained in a detailed statement (e.g. Data Clarification Form), reference to which must be documented on the relevant CRF page(s). Do not erase, overwrite, or use correction fluid on the original. All data printouts should be attached to the pertinent CRF pages.

The CRF for each subject must be reviewed and signed by the PI. This should be done as soon as possible after each subject completes the study. The PI will make the CRF pages available for review and collection at each scheduled monitoring visit. A clinical study monitor will review the CRF and compare the content versus the source data. The PI must retain the white photocopy CRF for his/her files. All CRF and other pertinent records are to be transferred to Dr. Brenner during and/or upon completion of the study.

The PI will retain a file copy of each completed CRF. In addition, the Investigator or designated colleague will provide access to the Medical Monitor for the periodic review of source documents (e.g., hospital and clinic records) to assure accuracy and completeness of the CRF. The investigator also must submit all incomplete CRF that reflect subject experience with the drug, including retrievable data on subjects who withdraw before completion of the study.

All clinical work conducted under this protocol is subject to GCP rules. This includes an inspection by RMC and/or Clalit Health services and/or health authority representatives at any time. The investigator will agree to the inspection of study-related records by RMC and/or Clalit Health services and/or health authority representatives.

## Data Management

Data Management services will be provided by Dr. Jaqueline Sulkes. All protocol-specified data documented on CRF or printouts will be entered via independent double-data entry into an electronic clinical database. Data captured in an electronic format will be compiled and reconciled with CRF data as applicable.

After the data have been entered and verified, various edit checks will be performed for the purpose of ensuring the accuracy, integrity, and validity of the database. These edit checks may include:

- Missing value checks
- Range checks
- Consistency checks
- Sequence checks
- Probabilistic checks
- Protocol adherence checks

Queries generated from these checks will be sent to the investigational site for resolution, and the database will be updated to reflect query resolutions as appropriate

Adverse events will be coded using the Medical Dictionary for Regulatory Activities (MedDRA, latest version available). Prior and concomitant medications will be coded according to the World Health Organization (WHO) Drug Dictionary.

# Study Administration

## Participating Centers

One center in Israel (RMC) will participate in the phase I part of this study; additional centers may participate in its phase II part.

## Study Personnel

Below is a list of key individuals and their roles that will contribute to this study:

Sponsor: Dr. Baruch Brenner

Medical Monitor: TBA

Study Monitor: TBA

## Required Documents Prior to Study Initiation

Prior to the start of this study, all pre-investigational requirements must be met by the Principal Investigator and study site. These may include:

- Appropriate local health authority documentation properly signed and dated by the required investigators.
- Signed copy (original) of the approved protocol.
- Completed and signed statement of investigator.
- A signed Clinical Trial Agreement
- Curriculum vitae and current medical licenses for the investigator and sub-investigators.
- IRB/IEC name and address; and membership list
- Letter of approval from the IRB/IEC for both protocol (identified by protocol title and number) and informed consent form (identified by protocol title and number).
- Copy of the IRB/IEC-approved written ICF to be used in the study (that has also been approved by the Sponsor).
- Provisions for direct access to source/data documents if necessary for trial-related monitoring, audits, IRB/IEC review, and regulatory inspection
- Name and location of the laboratory utilized for laboratory assays, and other facilities conducting tests, as well as a copy of the laboratory certificate and list of normal laboratory values.

In case a laboratory certification is not available, a written statement as to how the laboratory complies with quality assurance should be provided.

Upon satisfactory receipt of all required regulatory documents, Dr. Brenner will arrange that study drugs be delivered to the study site. Supply of all other study materials will be the responsibility of Dr. Baruch Brenner and/or designee. Subject entry should not begin until after the required regulatory documents are confirmed as received and the Investigator Meeting/Initiation Meeting has occurred. All personnel expected to be involved in the conduct of the study will undergo orientation to include review of study protocol, instructions for CRF completion, AE reporting, and overall responsibilities including those for drug accountability and study file maintenance.

The investigator and/or designee (study monitor) will prepare an Investigator's File. This file should be used for all trial related documents. The investigator will be responsible for keeping the Investigator's file updated and ensuring that all required documents are filed. The file will be inspected during monitoring visits.

## Clinical Trial Supplies

The PI will be responsible for the supplying, administrating, inventory, and accountability of all clinical trial supplies, exercising accepted medical and pharmaceutical practices. An accurate and timely record of the disposition of all clinical supplies must be maintained. The supplies and inventory record must be made available for inspection upon request. Upon completion or termination of the study the Investigator will keep the remaining clinical supplies along with a copy of the inventory record and a record of the clinical supplies returned. **Under no circumstances will the PI allow the study drugs to be used other than as directed by this protocol.**

Clinical trial supplies include, however, not limited to: CRF, lab supplies and study drugs.

## Investigator Site File

All documents required for the conduct of the study as specified in the ICH-GCP guidelines will be maintained by the Investigator in an orderly manner and made available for monitoring and/or auditing by the RMC, Clalit Health Services and regulatory agencies.

## Study Completion

This study is expected to end when all required subjects have been enrolled and the last subject has completed the study and the query resolution has been completed.

Data and materials that are required before the study can be considered complete and/or terminated are:

- Laboratory findings, clinical data, and all special test results from screening through the end of the follow-up period
- CRF (including correction forms) properly completed by appropriate study personnel and signed by the Investigator
- Completed Drug Accountability Records
- Statement of outcome for each serious adverse event reported
- Copies of protocol amendments and IRB/IEC as well as relevant health authority approval/notification (if applicable)

## Final Report

After completion or termination of the study the PI is required to submit a summary report to the IRB/IEC at RMC. Suggested inclusions in the report are: study objectives, methods (including any deviation from the study protocol), evaluation of the study results, observations by the investigator as to the safety and tolerance of the study drugs, and a discussion of all AEs and laboratory abnormalities. The level of detail in the report and the quality of the report should be suitable for submission to a peer-reviewed medical journal.

## Retention of Study Records

The PI will retain copies of the approved protocol, completed CRF, informed consent documents, relevant source documents, and all other supporting documentation related to the project for a minimum of two years. If the investigator is unable to retain the study documents for the required amount of time, RMC or designee must be informed of the individual who will be assuming this responsibility.

These files must be made available for inspection upon reasonable request by authorized representatives of RMC and/or Clalit Health Services and/or the relevant regulatory agencies.

## Confidentiality and Publication

Subject medical information obtained by the study is confidential and disclosure to third parties other than those noted below is prohibited. Throughout the study, all data will be identified only by the subject identification number, and where applicable, the subject’s initials.

At the subject's request, medical information may be given to his or her personal physician or other appropriate medical personnel responsible for his or her welfare. Personal physician will be notified by site personnel of subject participation in the study.

All information concerning this study that was not previously published is considered confidential information. This confidential information shall remain the property of Dr. Baruch Brenner and it shall not be disclosed, or submitted for presentation or publication to others without written permission of Dr. Baruch Brenner. The confidential information shall not be used except in the performance of this study.

# References

1. Kamangar F, Dores GM, Anderson WF. Patterns of cancer incidence, mortality, and prevalence across five continents: defining priorities to reduce cancer disparities in different geographic regions of the world. J Clin Oncol 2006; 24 (14):2137-2150.

2. Correa P. The epidemiology of gastric cancer. World J Surg 1991; 15 (2):228-234.

3. Franceschi S, Levi F, La Vecchia C. Epidemiology of gastric cancer in Europe. Eur J Cancer Prev 1994; 3 Suppl 2:5-10.

4. Kelsen DP. Adjuvant and neoadjuvant therapy for gastric cancer. Semin Oncol 1996; 23 (3):379-389.

5. Griffin JF, Smalley SR, Jewell W, Paradelo JC, Reymond RD, Hassanein RE, Evans RG. Patterns of failure after curative resection of pancreatic carcinoma. Cancer 1990; 66 (1):56-61.

6. Scartozzi M, Galizia E, Verdecchia L, Berardi R, Antognoli S, Chiorrini S, Cascinu S. Chemotherapy for advanced gastric cancer: across the years for a standard of care. Expert Opin Pharmacother 2007; 8 (6):797-808.

7. Rivera F, Vega-Villegas ME, Lopez-Brea MF. Chemotherapy of advanced gastric cancer. Cancer Treat Rev 2007; 33 (4):315-324.

8. Vanhoefer U, Rougier P, Wilke H, Ducreux MP, Lacave AJ, Van Cutsem E, Planker M, Santos JG, Piedbois P, Paillot B, Bodenstein H, Schmoll HJ, Bleiberg H, Nordlinger B, Couvreur ML, Baron B, Wils JA. Final results of a randomized phase III trial of sequential high-dose methotrexate, fluorouracil, and doxorubicin versus etoposide, leucovorin, and fluorouracil versus infusional fluorouracil and cisplatin in advanced gastric cancer: A trial of the European Organization for Research and Treatment of Cancer Gastrointestinal Tract Cancer Cooperative Group. J Clin Oncol 2000; 18 (14):2648-2657.

9. Kundel Y, Purim O, Figer A, Stemmer SM, Tichler T, Sulkes J, Sulkes A, Brenner B. Weekly infusional high-dose 5-fluorouracil and leucovorin and biweekly cisplatin: A convenient treatment option in advanced gastric cancer. Med Sci Monit 2008; 14 (4):CR190-195.

10. Van Cutsem E, Moiseyenko VM, Tjulandin S, Majlis A, Constenla M, Boni C, Rodrigues A, Fodor M, Chao Y, Voznyi E, Risse ML, Ajani JA. Phase III study of docetaxel and cisplatin plus fluorouracil compared with cisplatin and fluorouracil as first-line therapy for advanced gastric cancer: a report of the V325 Study Group. J Clin Oncol 2006; 24 (31):4991-4997.

11. Long DF, Repta AJ. Cisplatin: chemistry, distribution and biotransformation. Biopharm Drug Dispos 1981; 2 (1):1-16.

12. Loehrer PJ, Einhorn LH. Drugs five years later. Cisplatin. Ann Intern Med 1984; 100 (5):704-713.

13. Ishitsuka H. Capecitabine: preclinical pharmacology studies. Invest New Drugs 2000; 18 (4):343-354.

14. Van Cutsem E, Twelves C, Cassidy J, Allman D, Bajetta E, Boyer M, Bugat R, Findlay M, Frings S, Jahn M, McKendrick J, Osterwalder B, Perez-Manga G, Rosso R, Rougier P, Schmiegel WH, Seitz JF, Thompson P, Vieitez JM, Weitzel C, Harper P. Oral capecitabine compared with intravenous fluorouracil plus leucovorin in patients with metastatic colorectal cancer: results of a large phase III study. J Clin Oncol 2001; 19 (21):4097-4106.

15. Hoff PM, Ansari R, Batist G, Cox J, Kocha W, Kuperminc M, Maroun J, Walde D, Weaver C, Harrison E, Burger HU, Osterwalder B, Wong AO, Wong R. Comparison of oral capecitabine versus intravenous fluorouracil plus leucovorin as first-line treatment in 605 patients with metastatic colorectal cancer: results of a randomized phase III study. J Clin Oncol 2001; 19 (8):2282-2292.

16. Oshaughnessy JA, Blum J, Moiseyenko V, Jones SE, Miles D, Bell D, Rosso R, Mauriac L, Osterwalder B, Burger HU, Laws S. Randomized, open-label, phase II trial of oral capecitabine (Xeloda) vs. a reference arm of intravenous CMF (cyclophosphamide, methotrexate and 5-fluorouracil) as first-line therapy for advanced/metastatic breast cancer. Ann Oncol 2001; 12 (9):1247-1254.

17. Cunningham D, Starling N, Rao S, Iveson T, Nicolson M, Coxon F, Middleton G, Daniel F, Oates J, Norman AR. Capecitabine and oxaliplatin for advanced esophagogastric cancer. N Engl J Med 2008; 358 (1):36-46.

18. Cortes JE, Pazdur R. Docetaxel. J Clin Oncol 1995; 13 (10):2643-2655.

19. Tabernero J, Climent MA, Lluch A, Albanell J, Vermorken JB, Barnadas A, Anton A, Laurent C, Mayordomo JI, Estaun N, Losa I, Guillem V, Garcia-Conde J, Tisaire JL, Baselga J. A multicentre, randomised phase II study of weekly or 3-weekly docetaxel in patients with metastatic breast cancer. Ann Oncol 2004; 15 (9):1358-1365.

20. Schuette W, Nagel S, Blankenburg T, Lautenschlaeger C, Hans K, Schmidt EW, Dittrich I, Schweisfurth H, von Weikersthal LF, Raghavachar A, Reissig A, Serke M. Phase III study of second-line chemotherapy for advanced non-small-cell lung cancer with weekly compared with 3-weekly docetaxel. J Clin Oncol 2005; 23 (33):8389-8395.

21. O'Shaughnessy J, Miles D, Vukelja S, Moiseyenko V, Ayoub JP, Cervantes G, Fumoleau P, Jones S, Lui WY, Mauriac L, Twelves C, Van Hazel G, Verma S, Leonard R. Superior survival with capecitabine plus docetaxel combination therapy in anthracycline-pretreated patients with advanced breast cancer: phase III trial results. J Clin Oncol 2002; 20 (12):2812-2823.

22. Roth AD, Fazio N, Stupp R, Falk S, Bernhard J, Saletti P, Koberle D, Borner MM, Rufibach K, Maibach R, Wernli M, Leslie M, Glynne-Jones R, Widmer L, Seymour M, de Braud F. Docetaxel, cisplatin, and fluorouracil; docetaxel and cisplatin; and epirubicin, cisplatin, and fluorouracil as systemic treatment for advanced gastric carcinoma: a randomized phase II trial of the Swiss Group for Clinical Cancer Research. J Clin Oncol 2007; 25 (22):3217-3223.

23. Ferrara N. Role of vascular endothelial growth factor in regulation of physiological angiogenesis. Am J Physiol Cell Physiol 2001; 280 (6):C1358-1366.

24. Hurwitz H, Fehrenbacher L, Novotny W, Cartwright T, Hainsworth J, Heim W, Berlin J, Baron A, Griffing S, Holmgren E, Ferrara N, Fyfe G, Rogers B, Ross R, Kabbinavar F. Bevacizumab plus irinotecan, fluorouracil, and leucovorin for metastatic colorectal cancer. N Engl J Med 2004; 350 (23):2335-2342.

25. Sandler AB, Gray R, Brahmer J, Dowlati A, Schiller JH, Perry MC, Johnson DH. Randomized phase II/III Trial of paclitaxel (P) plus carboplatin (C) with or without bevacizumab (NSC # 704865) in patients with advanced non-squamous non-small cell lung cancer (NSCLC): An Eastern Cooperative Oncology Group (ECOG) Trial - E4599. Proc Am Soc Clin Oncol 2005; 23, 4.

26. Zon R, Miller KD, Wang M, Gralow J, Dickler M, Cobleigh M, Perez E, Shenkier T, Davidson N. A randomized phase III trial of paclitaxel with or without bevacizumab as first-line therapy for locally recurrent or metastatic breast cancer: Eastern cooperative oncology group trial E2100. Proc European Breast Cancer Conference, European J Cancer. 4 (Suppl): 47, 2006.

27. Miller KD. E2100: a phase III trial of paclitaxel versus paclitaxel/bevacizumab for metastatic breast cancer. Clin Breast Cancer 2003; 3 (6):421-422.

28. Shah MA, Ramanathan RK, Ilson DH, Levnor A, D'Adamo D, O'Reilly E, Tse A, Trocola R, Schwartz L, Capanu M, Schwartz GK, Kelsen DP. Multicenter phase II study of irinotecan, cisplatin, and bevacizumab in patients with metastatic gastric or gastroesophageal junction adenocarcinoma. J Clin Oncol 2006; 24 (33):5201-5206.

29. Sparano JA, Martino S, Jones V, et al. Phase III study of doxorubicin-cyclophosphamide followed by paclitaxel or docetaxel given every 3 weeks or weekly in patients with axillary node- positive or high-risk node-negative breast cancer: results of North American Breast Cancer Intergroup Trial E1199. Proc San Antonio Breast Cancer Symp 2005; 48.

30. Tebbutt N, Sourjina T, Strickland A, Van Hazel G, Ganju V, Gibbs D, Gebski V, Munro S, Cummins M. ATTAX: Randomised phase II study evaluating weekly docetaxel-based chemotherapy combinations in advanced esophago- gastric cancer, final results of an AGITG trial. Proc Am Soc Clin Oncol 2007; 25, 4528.

31. Grothe W, Hofheinz RD, Mantovani Loeffler L, Böhme J, Arnold D, Radestock U, Hochhaus A, Schmoll H. Phase I trial of docetaxel, oxaliplatin and capecitabine (TEX) in patients with metastatic gastric cancer. Proc Am Soc Clin Oncol 2006; 24, 14051.

32. Ajani J. Review of capecitabine as oral treatment of gastric, gastroesophageal, and esophageal cancers. Cancer 2006; 107 (2):221-231.

33. Lee JL, Kang YK. Capecitabine in the treatment of advanced gastric cancer. Future Oncol 2008; 4 (2):179-198.

34. Moehler M, Kanzler S, Geissler M, Raedle J, Ebert M, Scherubl H, Flieger D, et al. Irinotecan/Capecitabine versus Cisplatin/Capecitabine in advanced adenocarcinoma of the stomach or gastroesophageal junction: interim analysis of a German AIO phase II study. Proc Euro Soc Medic Oncol 2006; 17, 1093P.

35. Kang YK, Kang WK, Shin DB, Chen J, Xiong J, Wang JJ, Lichinitser M, et al. Randomized phase II trial of capecitabine/cisplatin (XP) vs. Continuous infusion of 5-FU/cisplatin (FP) as first-line therapy in patients (pts) with advanced gastric cancer (AGC): subgroup analyses confirm main efficacy findings. Proc Euro Soc Medic Oncol 2006; 17, 1072O.

36. Sawada N, Ishikawa T, Fukase Y, Nishida M, Yoshikubo T, Ishitsuka H. Induction of thymidine phosphorylase activity and enhancement of capecitabine efficacy by taxol/taxotere in human cancer xenografts. Clin Cancer Res 1998; 4 (4):1013-1019.

37. Thuss-Patience PC, Kretzschmar A, Dogan Y, Blau I, Pink D, Lebedinzew B, Micheel S, Dörken B, Reichardt P. Capecitabine and docetaxel for advanced gastric cancer. Proc Am Soc Clin Oncol 2006; 24, 4068.

38. Fondevila C, Metges JP, Fuster J, Grau JJ, Palacin A, Castells A, Volant A, Pera M. p53 and VEGF expression are independent predictors of tumour recurrence and survival following curative resection of gastric cancer. Br J Cancer 2004; 90 (1):206-215.

39. Karayiannakis AJ, Syrigos KN, Polychronidis A, Zbar A, Kouraklis G, Simopoulos C, Karatzas G. Circulating VEGF levels in the serum of gastric cancer patients: correlation with pathological variables, patient survival, and tumor surgery. Ann Surg 2002; 236 (1):37-42.

40. Enzinger PC, Fidias P, Meyerhardt J, Stuart K, Fuchs C, Huberman M, Goldstein R, Attawia M, Lawrence C, Zhu AX. Phase II study of bevacizumab and docetaxel in metastatic esophageal and gastric cancer. Proc Am Soc Clin Oncol Gastrointestinal Symposium 2006; 68.

41. Therasse P, Arbuck SG, Eisenhauer EA, Wanders J, Kaplan RS, Rubinstein L, Verweij J, Van Glabbeke M, van Oosterom AT, Christian MC, Gwyther SG. New guidelines to evaluate the response to treatment in solid tumors. European Organization for Research and Treatment of Cancer, National Cancer Institute of the United States, National Cancer Institute of Canada. J Natl Cancer Inst 2000; 92 (3):205-216.

42. Cancer Therapy Evaluation Program: Common Terminology Criteria for Adverse Events v3.0 (CTCAE), DCTD, NCI, NIH, DHHS. 2006; March 31, 2006.

###### APPENDICES

[Appendix A Study Flow Chart 52](#__RefHeading___Toc217022142)

[Appendix B Dose Escalation Scheme 53](#__RefHeading___Toc217022143)

[Appendix C Eastern Cooperative Oncology Group performance status 54](#__RefHeading___Toc217022144)

[Appendix D RECIST response criteria for solid tumors 55](#__RefHeading___Toc217022145)

[Appendix E Treatment interruptions/discontinuations 56](#__RefHeading___Toc217022146)

[Appendix F Declaration of Helsinki 61](#__RefHeading___Toc217022147)

Appendix A Study Flow Chart

| **STUDY PROCEDURES** | **Screening** | **BSLN Treatment Phase** | | | | **Early Termination** | **Follow-Up** |
| --- | --- | --- | --- | --- | --- | --- | --- |
|  |  | **Cycle 1** | | **Cycle n+1** | |  |  |
| Visit | 1 | 2 | 3 |  |  |  | Every 2 months |
| Visit Window (days) | + 7 | ± 3 days | | | |  | ± 7 days |
| Day in Cycle | -14 | 1 | 8 | 1 | 8 |  |  |
| **INVESTIGATOR ASSESSMENTS** |  |  |  |  |  |  |  |
| Obtain written informed consent | x |  |  |  |  |  |  |
| Assess inclusion/Exclusion criteria | x | x |  |  |  |  |  |
| Enroll subject & assign number |  | x |  |  |  |  |  |
| Obtain demographic & medical history | x |  |  |  |  |  |  |
| Conduct Physical Exam | x | x |  | x |  | x | x |
| ECG | x |  |  |  |  |  |  |
| Record Vital signs^1^ | x | x | x | x | x | x |  |
| Measure height & Weight | x |  |  |  |  |  |  |
| Record concomitant medication | x | x | x | x | x | x | x |
| Administer docetaxel |  | x | x | x | x |  |  |
| Administer cisplatin |  | x | x | x | x |  |  |
| Administer bevacizumab |  | x |  | x |  |  |  |
| Dispense capecitabine ^2^ |  | x |  | x |  |  |  |
| Perform drug accountability for capecitabine |  |  |  | x |  | x |  |
| Evaluate toxicity |  | x | x | x | x |  |  |
| Assess treatment compliance |  |  | x | x | x |  |  |
| Record Adverse Events |  | x | x | x | x | x |  |
| Assess tumor radiologically (CT scan) | x^3^ |  |  |  | x^4^ | x | Every three months |
| ECOG Performance Scale | x | x^7^ | x | x | x | x | x |
| Survival |  |  |  |  |  | x | x |
| **LABORATORY ASSESSMENTS** |  |  |  |  |  |  |  |
| Pregnancy test | x | x^6^ |  |  |  | x |  |
| Complete blood count (CBC) | x | x^6^ | x | x | x | x | x |
| Serum electrolytes (incl. creatinine) | x | x^6^ | x | x | x |  |  |
| Blood chemistry | x | x^6^ |  | x |  | x | x |
| Creatinine clearance | x | x^6^ |  |  |  |  |  |
| Tumor markers^5^ | x |  |  | x |  | x |  |
| Coagulation Markers | x |  |  |  |  |  |  |
| Urinalysis | x | x^6^ |  | x |  | x |  |

^1^Vital signs during the treatment period will be measured before and after each treatment and after the termination of bevacizumab infusion.

^2^Capecitabine is to be taken daily per os for 14 days in each cycle, followed by one week rest.

^3^CT within 28 days before baseline visit.

^4^Radiological evaluation will be conducted every 9 weeks. Tumor assessment will be done using the RECSIT criteria.

^5^At screening, CEA, CA-19.9 and CA-125 will be drawn. Subsequent tests will include only markers that were elevated at baseline.

^6^If the time period from screening to baseline exceeds 14 days, laboratory assessments and ECOG PS will be repeated

Appendix B Dose Escalation Scheme

The dose escalation scheme for the study regimen is based on the toxicities observed during the first two cycles of treatment. The starting dose level will be defined as level 0.

A minimum of three (3) patients must be followed for at least two (2) complete cycles (i.e. 6 weeks) of treatment before the trial can escalate to the next dose level. If none of the 3 patients experience DLT as described above then new patients will be entered at the next higher dose level. The dose level is escalated in successive cohorts of patients as long as no DLT is observed. If one instance of DLT is observed among the initial 3 patients treated at a dose level, an additional 3 patients must be treated at that dose level with no further DLT in order that dose escalation may proceed. If 2 instances of DLT are observed at a dose level, the MTD has been surpassed. Once the criteria for DLT for the study regimen have been met, we will decrease the regimen's dose to one dose level immediately below. At the lower dose level 3 more patients will be treated, unless 6 patients have already been treated at that dose level with < 1 DLT observed, in which case that dose level will already be determined as the MTD for the regimen. If less than 6 patients have been treated at the lower dose level, additional patients, to a total number of at least 6 patients, will be treated at that dose level. If no excessive toxicity is observed (i.e. < 1 DLT), is observed this new dose level will be declared the MTD of the regimen. If we do observe excessive toxicity, we will again reduce the dose back to one dose level immediately below. This process will be repeated until the MTD is identified. In the MTD, no more than 1 out of 6 or more patients will experience DLT. The MTD will serve as the dose level used in the phase II part of the study. If two or more patients in dose level –1 (one level immediately below the initial dose level) experience a DLT, the study will be closed early and the regimen will be considered non-tolerable.

In the regimen used in this study the initial doses of Capecitabine (1600 mg/m^2^/d) and Bevacizumab (7.5 mg/kg) will not escalate whereas the doses of docetaxel and cisplatin will be gradually increased, according to the predefined dose elevation schema. For that purpose, 4 dose levels, including level -1 in case of excessive toxicity in the initial dose level are planned, as follows:

| Dose Level | Cisplatin | Docetaxel |
| --- | --- | --- |
| -1 | 25 mg/m^2^ | 30 mg/m^2^ |
| 0 | 30 mg/m^2^ | 30 mg/m^2^ |
| 1 | 30 mg/m^2^ | 35 mg/m^2^ |
| 2 | 35 mg/m^2^ | 35 mg/m^2^ |

Appendix C Eastern Cooperative Oncology Group performance status

| **Eastern Cooperative Oncology Group (Zubrod-ECOG)^1,2^** | |
| --- | --- |
| **Description** | **Grade** |
| Fully active, able to carry on all pre-disease activities without restriction. | 0 |
| Restricted in physically strenuous activity but ambulatory and able to carry out work of a light or sedentary nature e.g. light house work, office work. | 1 |
| Ambulatory and capable of all self care but unable to carry out any work activities. Up and about more than 50% of waking hours. | 2 |
| Capable of only limited self care, confirmed to bed or chair more than 50% of waking hours. | 3 |
| Completely disabled. Cannot carry on any self care. Totally confined to bed or chair. | 4 |
| 1 Zubrod, C.G., et al. *Appraisal of Methods for the Study of Chemotherapy of Cancer in Man*. Journal of Chronic Diseases, 11:7-33, 1960.  2 Oken, M.M., et al. *Toxicity and response criteria of the Eastern Cooperative Oncology Group*. Am J Clin Oncol (CCT) 5: 649-55, 1982 | |

######

**Appendix D RECIST response criteria for solid tumors**

Objective Response Criteria (RECIST)

**Complete Response**: Disappearance of all clinical and radiological evidence of tumor (both *target* and *nontarget*) including normalization of elevated tumor markers at baseline, if documented. The patient must be free of all tumor-related symptoms. Complete Response must be confirmed at a second tumor assessment not less than 28 days apart from the assessment at which CR was observed.

**Partial Response**: At least a 30% decrease in the sum of longest diameter (LD) of target lesions taking as reference the baseline sum LD. Partial Response must be confirmed at a second tumor assessment not less than 28 days apart from the assessment at which PR was observed.

**Stable Disease:** Steady state of disease. Neither sufficient shrinkage to qualify for PR nor sufficient increase to qualify for PD. Stable disease must be documented to be present at least 28 days from the start of the therapy. There may be no appearance of new lesions for this category.

**Progressive Disease**: At least a 20% increase in the sum of LD of measured lesions taking as references the smallest sum LD recorded since the treatment started. Appearance of new lesions will also constitute PD. In exceptional circumstances, unequivocal progression of nontarget lesions may be accepted as evidence of disease progression.

Appendix E Drug-specific treatment interruptions/discontinuations

**Capecitabine**

If any grade > 2 non-hematological toxicity occurs which can not be attributed solely to other drug, INTERRUPT CAPECITABINE IMMEDIATELY and follow instructions below for further actions. The recommendations found in this section for dose adjustments for capecitabine should be followed for those toxicities usually considered to be related to capecitabine treatment. Thus, for example, neurotoxicity (CDDP), or hypertension and proteinuria (bevacizumab) do not result in a dose reduction for capecitabine.

**Note: capecitabine treatment interruptions are regarded as lost treatment days and the**

**planned treatment schedule should be maintained. Missed doses due to treatment**

**interruptions must not be replaced.**

If serum creatinine increases during treatment to > 2.0 mg/dl, treatment should be discontinued.

Hyperbilirubinemia: administration of capecitabine should be interrupted if treatment-related elevations in bilirubin of > 3.0 x ULN (Grade 3) occur. Treatment may be resumed when bilirubin decreases to ≤ 3.0 x ULN (Grade <2).

Treatment-related elevations in hepatic aminotransferases (ALT, AST) and alkaline

phosphatase (ALP) the guidance in [Table 19] is consistent with the management

outlined in Section 7.5.2.

Grade ≥ 2 Diarrhea

Capecitabine can induce diarrhea, which can sometimes be severe. Patients with severe diarrhea should be carefully monitored and, if they become dehydrated, should be given fluid and electrolyte replacement. If Grade >2 diarrhea occurs, administration of capecitabine should be immediately interrupted until the diarrhea resolves or decreases in intensity to Grade ≤ 1. Following the second occurrence of Grade >2 toxicity, subsequent doses of capecitabine should be decreased. Standard antidiarrhea treatments (e.g. loperamide) should be initiated, as medically appropriate, as early as possible. Capecitabine can not be re-started until diarrhea has resolved to Grade <1 and no loperamide has been given for 24 hours.

Grade ≥ 2 Nausea/Vomiting

Capecitabine can induce nausea or vomiting. If Grade >2 nausea and/or vomiting occurs, administration of capecitabine should be immediately interrupted until these symptoms resolve or decrease in intensity to Grade ≤ 1. Treat symptomatically. For prophylaxis, the patients must be supplied with oral anti-emetics in order to treat themselves in case nausea or vomiting occurs at home. The administration of oral metoclopramide is recommended for capecitabine-induced nausea (the use of 5-HT3 antagonists is at the discretion of the investigator). Adequate secondary therapeutic and prophylactic treatment has to be initiated once nausea or vomiting has occurred. If nausea/vomiting recur despite adequate prophylaxis, then dose modifications should also be made (Table 6.6).

Grade ≥ 2 Hand/Foot Syndrome

Hand-and-foot syndrome (palmar-plantar erythrodysesthesia or chemotherapy-induced acral erythema) is a cutaneous toxicity with a severity range of Grades 1 to 3 as shown:

Grade 1: skin changes or dermatitis without pain (e.g. erythema, peeling).

Grade 2: skin changes with pain, not interfering with function.

Grade 3: skin changes with pain, interfering with function.

If Grade >2 hand-foot syndrome occurs, administration of capecitabine should be immediately interrupted until the event resolves or decreases in intensity to Grade ≤ 1. Subsequent doses of capecitabine should be administered as per Table 6.6. Hand-foot syndrome should be treated symptomatically (i.e. use of emollients is recommended). The use of vitamin B6 is not permitted for symptomatic or secondary prophylactic treatment of hand-foot syndrome; impaired efficacy has been reported with concomitant use of vitamin B6 and cisplatin.

Grade ≥ 2 Stomatitis

If Grade >2 stomatitis occurs, administration of capecitabine should be immediately interrupted until the event resolves or decreases in intensity to Grade ≤ 1. Treat symptomatically. Subsequent doses of capecitabine should be administered as per Table 6.6.

Cardiac toxicity

For Grade ≥ 2 cardiac toxicity which is attributable to capecitabine, patients will be permanently discontinued from capecitabine therapy.

**Bevacizumab**

Life threatening toxicities seen with bevacizumab to date have been hemorrhage, thromboembolic events and gastro-intestinal perforation. Less severe toxicities include proteinuria, hypertension, wound healing complications, diarrhoea, nausea, pain, asthenia and epistaxis. Because of the long half-life of bevacizumab (20 days), the discontinuation of treatment in case of toxicity is not expected to influence its short-term clinical evolution and hence, the management of adverse events is based on institution of adequate treatment. This section provides guidance for treatment modifications due to toxicities attributable to bevacizumab.

**No dose reduction of bevacizumab is foreseen for an individual patient**. In general, toxicity attributable to bevacizumab will require bevacizumab treatment to be held or permanently discontinued. **Missed doses of bevacizumab will not be made up**.

Any patient who develops any one of the following toxicities attributable to bevacizumab should not receive further bevacizumab:

– Gastrointestinal perforation,

– Arterial thromboembolic events,

– Grade 3/4 haemorrhagic events,

– Symptomatic Grade 4 venous thromboembolic events,

– Grade 4 hypertension (hypertensive crisis) and hypertensive encephalopathy

– Grade 4 proteinuria (nephrotic syndrome).

– Allergic/ hypersensitivity reactions (any grade).

– Grade 3/4 Left Ventricular Systolic Dysfunction.

Hemorrhage If a Grade 3/4 bleeding occurs, appropriate treatment should be instituted and bevacizumab treatment will be discontinued permanently.

Thrombosis/Embolism Patients who develop the following Grades of thrombosis/embolism must discontinue bevacizumab and the following action is recommended:

• Bevacizumab should be permanently discontinued in patients who develop any Grade of arterial thromboembolic event.

• Venous thromboembolic event – Grade 3 or incidentally discovered pulmonary embolus (first occurrence): hold bevacizumab for 2 weeks. Bevacizumab may be resumed after initiation of therapeutic-dose anticoagulant therapy as soon as all of the following criteria are met:

– The patient must be on a stable dose of anticoagulant and, if on an oral coumarin derivative, have an INR within the target range (usually between 2 and 3) prior to restarting bevaczumab,

– The patient must not have had a Grade >3 haemorrhagic event since entering the study.

• Symptomatic Grade 4 venous thromboembolic event (first occurrence) – permanently discontinue bevacizumab.

Hypertension

Patients should be monitored for the development or worsening of hypertension via frequent blood pressure measurement. Blood pressure measurements should be taken after the patient has been in a resting position for ≥ 5 minutes. Repeated measurement of blood pressure for verification should be undertaken if the initial reading is ≥ 140 mmHg systolic and/or ≥ 90 mmHg diastolic blood pressure. All toxicity will be graded according to CTCAE v 3.0 guidelines:

• Grade 1 hypertension: Asymptomatic, transient (< 24 hrs) increase by > 20 mmHg (diastolic) or to > 150/100 mmHg if previously within normal limits. Intervention not indicated.

• Grade 2 hypertension: Recurrent or persistent (> 24 hr) or symptomatic increase by > 20 mmHg (diastolic) or to > 150/100 mmHg if previously within normal limits. Monotherapy of anti-hypertensive may be indicated. Once controlled to <150/100 mmHg, patients may continue bevacizumab therapy.

• Grade 3 hypertension: Requiring > 1 anti-hypertensive or more intensive therapy than previously. Bevacizumab should be withheld for persistent or symptomatic hypertension and should be permanently discontinued if hypertension is not controlled.

• Grade 4 hypertension: Life threatening consequence (e.g. hypertensive crisis). Occurrence of Grade 4 hypertension should lead to permanent discontinuation of bevacizumab. All doses of anti-hypertensive medicines should be recorded at all visits.

Proteinuria Patients will have a dipstick urinalysis according to the SoA unless proteinuria has been determined by 24-hour urine collection. All toxicity will be graded according to CTCAE v 3.0 guidelines. Proteinuria assessment and adjustment of bevacizumab administration for proteinuria should be in line with recommendation as defined below:

**First occurrence of proteinuria:**

• 1+ (dipstick): Administer bevacizumab as scheduled, NO additional evaluation is required.

• ≥2+ (dipstick): If 2+ or 3+ (dipstick) administer bevacizumab as scheduled and collect 24-hour urine to determine the total protein within 3 days prior to the next scheduled dose. If 4+ (dipstick): withhold bevacizumab and collect 24-hour urine to determine the total protein within 3 days prior to the next scheduled dose:

- 24-hour proteinuria ≤ 2 g: administer bevacizumab as scheduled.

- 24-hour proteinuria > 2 g: bevacizumab will be withheld pending next 24-hour total protein.

- Repeat 24-hour urine protein ≤ 2 g: administer bevacizumab as scheduled. 24-hour protein should be further monitored prior to each administration of bevacizumab until it has decreased to ≤ 1 g/24-hour.

- Repeat 24-hour urine protein > 2 g: bevacizumab dose should be withheld until 24-hour protein has decreased to ≤ 2 g. 24-hour protein should be further monitored prior to each administration of bevacizumab until it has decreased to ≤ 1 g/24-hour.

**Second and subsequent occurrence of ≥ 2+ proteinuria (dipstick):**

• 2+ (dipstick): administer bevacizumab as scheduled, NO additional evaluation is required.

• 3+ (dipstick): administer bevacizumab as scheduled and collect 24-hour urine to determine the total protein within 3 days prior to the next scheduled dose.

• 4+ (dipstick): withhold bevacizumab and collect 24-hour urine to determine the total protein within 3 days prior to the next scheduled dose.

• 24-hour proteinuria ≤ 2 g: administer bevacizumab as scheduled.

• 24-hour proteinuria > 2 g: Bevacizumab treatment should be withheld pending next 24 hour total protein.

- Repeat 24-hour urine protein ≤ 2 g: administer bevacizumab as schedule. 24-hour protein should be further monitored prior to each administration of bevacizumab until it has decreased to ≤ 1 g/24-hour.

- Repeat 24-hour urine protein > 2 g: bevacizumab dose should be withheld until 24-hour protein has decreased to ≤ 2 g. 24-hour protein should be further monitored prior to each administration of bevacizumab until it has decreased to ≤ 1 g/24-hour.

• Nephrotic syndrome (Grade 4, CTCAE v 3.0): Discontinue bevacizumab treatment

Gastro-intestinal Perforations

If a gastro-intestinal perforation occurs, appropriate treatment should be instituted and bevacizumab treatment will be discontinued permanently.

Wound Healing Complications

Bevacizumab therapy should not be initiated earlier than 28 days following major surgery or until the surgical wound is fully healed. In patients who experience wound healing complications during bevacizumab treatment, bevacizumab should be withheld until the wound is fully healed. Bevacizumab therapy should be withheld for elective surgery.

Fistula or intra-abdominal abscess

Patients who develop a fistula or intra-abdominal abscess should discontinue bevacizumab. However, it is at the investigator’s discretion to continue after resolution of the findings in selected patients.

Infusion-related or allergic reactions

In clinical studies, infusion reactions with the first dose of bevacizumab were uncommon (< 3%) and severe reactions occurred in 0.2% of patients. If mild (grade <2) infusion-related reactions (e.g., fever, chills, headache, nausea) occur, pre-medications should be given with the next dose and the infusion time will be increased by 30 ± 10 min. For patients with grade 3 infusion-related reactions, the bevacizumab infusion should be stopped and not restarted on that day. Adequate information on rechallenge of bevacizumab is not available. At the physician's discretion, bevacizumab may be permanently discontinued or re-instituted with pre-medications and at a rate of 90 ± 15 minutes. If the reaction occurred at the 90-minute rate, bevacizumab should be permanently discontinued. Similarly, in case of any doubt, bevacizumab should be permanently discontinued. When bevacizumab is re-instituted, the patient should be monitored, per physician's usual practice, for duration comparable to duration of reaction. For patients with grade 4 infusion-related reactions, bevacizumab should be permanently discontinued.

Anaphylaxis is defined as vascular collapse and shock (blood pressure <90 mm Hg that is unresponsive to IV fluids) believed to be allergic in origin, with or without antecedent respiratory distress and occurring within 30 minutes of initiation of bevacizumab infusion. Cutaneous manifestations include pruritus, urticaria, or angioedema. **Patients experiencing any grade of allergic reactions should permanently discontinue bevacizumab.**

Appendix F Declaration of Helsinki

Adopted by the 18th WMA General Assembly, Helsinki, Finland, June 1964, and amended by the:
29th WMA General Assembly, Tokyo, Japan, October 1975
35th WMA General Assembly, Venice, Italy, October 1983
41st WMA General Assembly, Hong Kong, September 1989
48th WMA General Assembly, Somerset West, Republic of South Africa, October 1996
52nd WMA General Assembly, Edinburgh, Scotland, October 2000
53th WMA General Assembly, Washington 2002 (Note of Clarification on paragraph 29 added)
55th WMA General Assembly, Tokyo 2004 (Note of Clarification on Paragraph 30 added)
59th WMA General Assembly, Seoul, October 2008

1. INTRODUCTION
   1. The World Medical Association (WMA) has developed the Declaration of Helsinki as a statement of ethical principles for medical research involving human subjects, including research on identifiable human material and data.

The Declaration is intended to be read as a whole and each of its constituent paragraphs should not be applied without consideration of all other relevant paragraphs.

- 1. Although the Declaration is addressed primarily to physicians, the WMA encourages other participants in medical research involving human subjects to adopt these principles.
  2. It is the duty of the physician to promote and safeguard the health of patients, including those who are involved in medical research. The physician's knowledge and conscience are dedicated to the fulfilment of this duty.
  3. The Declaration of Geneva of the WMA binds the physician with the words, "The health of my patient will be my first consideration," and the International Code of Medical Ethics declares that, "A physician shall act in the patient's best interest when providing medical care."
  4. Medical progress is based on research that ultimately must include studies involving human subjects. Populations that are underrepresented in medical research should be provided appropriate access to participation in research.
  5. In medical research involving human subjects, the well-being of the individual research subject must take precedence over all other interests.
  6. The primary purpose of medical research involving human subjects is to understand the causes, development and effects of diseases and improve preventive, diagnostic and therapeutic interventions (methods, procedures and treatments). Even the best current interventions must be evaluated continually through research for their safety, effectiveness, efficiency, accessibility and quality.
  7. In medical practice and in medical research, most interventions involve risks and burdens.
  8. Medical research is subject to ethical standards that promote respect for all human subjects and protect their health and rights. Some research populations are particularly vulnerable and need special protection. These include those who cannot give or refuse consent for themselves and those who may be vulnerable to coercion or undue influence.
  9. Physicians should consider the ethical, legal and regulatory norms and standards for research involving human subjects in their own countries as well as applicable international norms and standards. No national or international ethical, legal or regulatory requirement should reduce or eliminate any of the protections for research subjects set forth in this Declaration.

1. BASIC PRINCIPLES FOR ALL MEDICAL RESEARCH
   1. It is the duty of physicians who participate in medical research to protect the life, health, dignity, integrity, right to self-determination, privacy, and confidentiality of personal information of research subjects.
   2. Medical research involving human subjects must conform to generally accepted scientific principles, be based on a thorough knowledge of the scientific literature, other relevant sources of information, and adequate laboratory and, as appropriate, animal experimentation. The welfare of animals used for research must be respected.
   3. Appropriate caution must be exercised in the conduct of medical research that may harm the environment.
   4. The design and performance of each research study involving human subjects must be clearly described in a research protocol. The protocol should contain a statement of the ethical considerations involved and should indicate how the principles in this Declaration have been addressed. The protocol should include information regarding funding, sponsors, institutional affiliations, other potential conflicts of interest, incentives for subjects and provisions for treating and/or compensating subjects who are harmed as a consequence of participation in the research study. The protocol should describe arrangements for post-study access by study subjects to interventions identified as beneficial in the study or access to other appropriate care or benefits.
   5. The research protocol must be submitted for consideration, comment, guidance and approval to a research ethics committee before the study begins. This committee must be independent of the researcher, the sponsor and any other undue influence. It must take into consideration the laws and regulations of the country or countries in which the research is to be performed as well as applicable international norms and standards but these must not be allowed to reduce or eliminate any of the protections for research subjects set forth in this Declaration. The committee must have the right to monitor ongoing studies. The researcher must provide monitoring information to the committee, especially information about any serious adverse events. No change to the protocol may be made without consideration and approval by the committee.
   6. Medical research involving human subjects must be conducted only by individuals with the appropriate scientific training and qualifications. Research on patients or healthy volunteers requires the supervision of a competent and appropriately qualified physician or other health care professional. The responsibility for the protection of research subjects must always rest with the physician or other health care professional and never the research subjects, even though they have given consent.
   7. Medical research involving a disadvantaged or vulnerable population or community is only justified if the research is responsive to the health needs and priorities of this population or community and if there is a reasonable likelihood that this population or community stands to benefit from the results of the research.
   8. Every medical research study involving human subjects must be preceded by careful assessment of predictable risks and burdens to the individuals and communities involved in the research in comparison with foreseeable benefits to them and to other individuals or communities affected by the condition under investigation.
   9. Every clinical trial must be registered in a publicly accessible database before recruitment of the first subject.
   10. Physicians may not participate in a research study involving human subjects unless they are confident that the risks involved have been adequately assessed and can be satisfactorily managed. Physicians must immediately stop a study when the risks are found to outweigh the potential benefits or when there is conclusive proof of positive and beneficial results.
   11. Medical research involving human subjects may only be conducted if the importance of the objective outweighs the inherent risks and burdens to the research subjects.
   12. Participation by competent individuals as subjects in medical research must be voluntary. Although it may be appropriate to consult family members or community leaders, no competent individual may be enrolled in a research study unless he or she freely agrees.
   13. Every precaution must be taken to protect the privacy of research subjects and the confidentiality of their personal information and to minimize the impact of the study on their physical, mental and social integrity.
   14. In medical research involving competent human subjects, each potential subject must be adequately informed of the aims, methods, sources of funding, any possible conflicts of interest, institutional affiliations of the researcher, the anticipated benefits and potential risks of the study and the discomfort it may entail, and any other relevant aspects of the study. The potential subject must be informed of the right to refuse to participate in the study or to withdraw consent to participate at any time without reprisal. Special attention should be given to the specific information needs of individual potential subjects as well as to the methods used to deliver the information. After ensuring that the potential subject has understood the information, the physician or another appropriately qualified individual must then seek the potential subject's freely-given informed consent, preferably in writing. If the consent cannot be expressed in writing, the non-written consent must be formally documented and witnessed.
   15. For medical research using identifiable human material or data, physicians must normally seek consent for the collection, analysis, storage and/or reuse. There may be situations where consent would be impossible or impractical to obtain for such research or would pose a threat to the validity of the research. In such situations the research may be done only after consideration and approval of a research ethics committee.
   16. When seeking informed consent for participation in a research study the physician should be particularly cautious if the potential subject is in a dependent relationship with the physician or may consent under duress. In such situations the informed consent should be sought by an appropriately qualified individual who is completely independent of this relationship.
   17. For a potential research subject who is incompetent, the physician must seek informed consent from the legally authorized representative. These individuals must not be included in a research study that has no likelihood of benefit for them unless it is intended to promote the health of the population represented by the potential subject, the research cannot instead be performed with competent persons, and the research entails only minimal risk and minimal burden.
   18. When a potential research subject who is deemed incompetent is able to give assent to decisions about participation in research, the physician must seek that assent in addition to the consent of the legally authorized representative. The potential subject's dissent should be respected.
   19. Research involving subjects who are physically or mentally incapable of giving consent, for example, unconscious patients, may be done only if the physical or mental condition that prevents giving informed consent is a necessary characteristic of the research population. In such circumstances the physician should seek informed consent from the legally authorized representative. If no such representative is available and if the research cannot be delayed, the study may proceed without informed consent provided that the specific reasons for involving subjects with a condition that renders them unable to give informed consent have been stated in the research protocol and the study has been approved by a research ethics committee. Consent to remain in the research should be obtained as soon as possible from the subject or a legally authorized representative.
   20. Authors, editors and publishers all have ethical obligations with regard to the publication of the results of research. Authors have a duty to make publicly available the results of their research on human subjects and are accountable for the completeness and accuracy of their reports. They should adhere to accepted guidelines for ethical reporting. Negative and inconclusive as well as positive results should be published or otherwise made publicly available. Sources of funding, institutional affiliations and conflicts of interest should be declared in the publication. Reports of research not in accordance with the principles of this Declaration should not be accepted for publication.
2. ADDITIONAL PRINCIPLES FOR MEDICAL RESEARCH COMBINED WITH MEDICAL CARE
   1. The physician may combine medical research with medical care only to the extent that the research is justified by its potential preventive, diagnostic or therapeutic value and if the physician has good reason to believe that participation in the research study will not adversely affect the health of the patients who serve as research subjects.
   2. The benefits, risks, burdens and effectiveness of a new intervention must be tested against those of the best current proven intervention, except in the following circumstances:
   - The use of placebo, or no treatment, is acceptable in studies where no current proven intervention exists; or
   - Where for compelling and scientifically sound methodological reasons the use of placebo is necessary to determine the efficacy or safety of an intervention and the patients who receive placebo or no treatment will not be subject to any risk of serious or irreversible harm. Extreme care must be taken to avoid abuse of this option.
   1. At the conclusion of the study, patients entered into the study are entitled to be informed about the outcome of the study and to share any benefits that result from it, for example, access to interventions identified as beneficial in the study or to other appropriate care or benefits.
   2. The physician must fully inform the patient which aspects of the care are related to the research. The refusal of a patient to participate in a study or the patient's decision to withdraw from the study must never interfere with the patient-physician relationship.
   3. In the treatment of a patient, where proven interventions do not exist or have been ineffective, the physician, after seeking expert advice, with informed consent from the patient or a legally authorized representative, may use an unproven intervention if in the physician's judgement it offers hope of saving life, re-establishing health or alleviating suffering. Where possible, this intervention should be made the object of research, designed to evaluate its safety and efficacy. In all cases, new information should be recorded and, where appropriate, made publicly available.

1. The study is supported by Roche Pharmaceuticals (Israel) Ltd and Sanofi-Aventis Pharmaceuticals. [↑](#footnote-ref-2)
